# Chiral supramolecular architecture of stable transmembrane pores formed by an α-helical antibiotic peptide in the presence of lyso-lipids

**DOI:** 10.1038/s41598-020-61526-w

**Published:** 2020-03-13

**Authors:** Erik Strandberg, David Bentz, Parvesh Wadhwani, Anne S. Ulrich

**Affiliations:** 1 Karlsruhe Institute of Technology (KIT), Institute of Biological Interfaces (IBG-2), POB 3640, 76021 Karlsruhe, Germany; 2 KIT, Institute of Organic Chemistry, Fritz-Haber-Weg 6, 76131 Karlsruhe, Germany

**Keywords:** Biophysics, Membrane biophysics, Membrane structure and assembly, Molecular biophysics, Structural biology, NMR spectroscopy

## Abstract

The amphipathic α-helical antimicrobial peptide MSI-103 (aka KIA21) can form stable transmembrane pores when the bilayer takes on a positive spontaneous curvature, e.g. by the addition of lyso-lipids. Solid-state ^31^P- and ^15^N-NMR demonstrated an enrichment of lyso-lipids in these toroidal wormholes. Anionic lyso-lipids provided additional stabilization by electrostatic interactions with the cationic peptides. The remaining lipid matrix did not affect the nature of the pore, as peptides maintained the same orientation independent of lipid charge, and a change in membrane thickness did not considerably affect their tilt angle. Under optimized conditions (i.e. in the presence of lyso-lipids and appropriate bilayer thickness), stable and well-aligned pores could be obtained for solid-state ^2^H-NMR analysis. These data revealed for the first time the complete 3D alignment of this representative amphiphilic peptide in fluid membranes, which is compatible with either monomeric helices as constituents, or left-handed supercoiled dimers as building blocks from which the overall toroidal wormhole is assembled.

## Introduction

Membrane-active antimicrobial peptides (AMPs) can kill microorganisms by permeabilizing the cell membrane. They are attracting much attention as potential new antibiotics against the increasingly common multidrug-resistant bacterial strains^1–4^. While it is clear that these peptides can target a wide range of microorganisms, the molecular mechanisms of membrane permeabilization are not yet fully characterized. That is because the membrane damaging step is rapid, pores tend to be highly dynamic, and thereby the systems evade structural analysis with high resolution. Cationic amphipathic α-helices constitute one of the most common types of AMPs. Among other models, they have been proposed to form transmembrane pores, leading to leakage through the lipid bilayer and thereby cell death^5–9^. A representative AMP is MSI-103 (also called KIA21), a designer-made peptide with a repeated heptamerical sequence found in PGLa [GMASKAGAIAGKIAKVALKAL-NH_2_], which is a natural AMP found in the skin of the frog *Xenopus laevis*^10,11^. MSI-103, with the 21-mer sequence (KIAGKIA)_3_-NH_2_, is a highly active AMP and has been well characterized with regard to its membrane interactions using various biological assays and biophysical methods^12–14^.

The membrane-bound structure of MSI-103 has been studied extensively using solid-state NMR (SSNMR) and other methods^14–19^. One important conclusion from SSNMR studies of MSI-103 in different lipid systems is that the helix orientation in the membrane depends critically on the spontaneous curvature of the constituent lipids^15,19,20^. Under most conditions the amphiphilic helix binds flat onto the bilayer surface, but an upright transmembrane insertion is promoted by lipids with a positive spontaneous curvature, such as lyso-lipids with a large polar head group and a single acyl chain^20^.

It has recently been shown that peptide “rulers” derived from MSI-103 have a length-dependent mechanism of action^14,21^. Using a set of peptides with different lengths from 14 to 28 amino acids, called KIA peptides (as they are based on the repeat sequence KIAGKIA), it was found that the helix must be long enough to span the hydrophobic part of the membrane in order to form pores and cause permeabilization^14,21^. Solid-state ^15^N-NMR studies of KIA peptides showed that – under equilibrium conditions of a fully hydrated aligned NMR sample in the liquid crystalline state – all peptides lie flat on the membrane surface in bilayers made of lipids containing unsaturated acyl chains, such as POPC and POPG^18^. However, they were found to be inserted in a transmembrane orientation in lipid systems with a highly positive spontaneous curvature, such as DMPC and lyso-MPC^18^. Lyso-lipids thus offer the unique opportunity to establish conditions where the peptides become assembled into stable transmembrane pores of the toroidal wormhole type. These trapped pores can now be characterized in detail by solid-state ^2^H-NMR, under conditions that presumably reflect the instantaneous moment of transient pore formation.

Here, we have studied the detailed structure and deduced the superstructure of pores formed by MSI-103 in bilayers containing lyso-lipids, using solid state NMR on macroscopically aligned multilayered membrane samples. This is the method of choice to study the detailed conformation, orientation, dynamics and self-assembly of membrane-active peptides in a quasi-native environment^22–30^. Here, we used ^15^N- and ^2^H-NMR on MSI-103 that was either specifically labeled with ^15^N on the amide in the backbone, or deuterium labeled with Ala-d_3_ at several individual positions, to extend and refine previous NMR-derived models of the peptide pore^14,16,18,19^. The effects of varying the amount and type of lyso-lipid, besides varying peptide concentration, were systematically examined to obtain optimal conditions that would promote a stable pore. The influence of particular lipid properties on the orientation of the peptide in the pore was also investigated. The acyl chain length of both, the lipid matrix and of the lyso-lipid, was systematically varied, and the effect of charge on the lipid matrix and the lyso-lipid was also examined.

**Figure 1:**
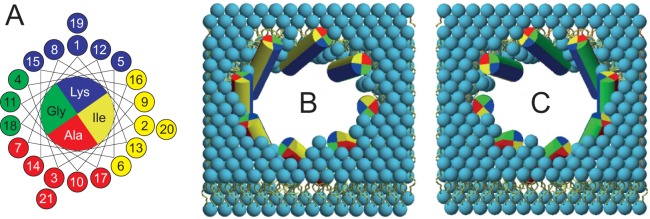
(**A**) Helical wheel projection of MSI-103 (also known as KIA21). Residues are color-coded as follows: blue for Lys, yellow for Ile, red for Ala, and green for Gly. The number in each circle is the residue number. (**B**,**C**) The peptide helices are shown as cylinders, color-coded according to (**A**), and all are viewed from the N-terminus. Lipids have light blue headgroups and yellow acyl chains. Tilted helices are expected to assemble into a chiral super-structure with either a (**B**) left-handed or (**C**) right-handed bundle. The two models illustrated here are constructed based on our previous knowledge of the helix tilt angle, and such that the charged Lys residues are pointing into the pore, but the number of monomers represents only an educated guess. In order to decide whether either a left-handed (**B**) or a right-handed (**C**) bundle is formed, the correct peptide orientation in the pore needs to be determined from the azimuthal rotation angle.

Of particular interest is the precise orientation of the amphipathic peptide in the pore. An approximate helix tilt angle had been previously estimated from ^15^N-NMR^18^, so here we used ^2^H-NMR to obtain a more accurate value for the tilt. With this method, it was also possible to determine for the first time the azimuthal angle, which describes the characteristic rotational angle of the amphiphilic peptide around its helix axis. From these two angles, the complete 3D orientation of the helix backbone in a stable pore could be characterized, including its dynamical behavior. We had recently proposed that the tilted peptides, which constitute the pore, are arranged as a kind of iris diaphragm^18^. The inside of the pore should be lined with polar residues, so one would expect the Lys residues to point towards the center, as illustrated in Fig. 1. From geometric considerations, such a pore must have a supramolecular chirality^31^, as the intrinsically chiral α-helices can be tilted either in a right-handed or in a left-handed sense (Fig. 1). By obtaining here the full orientation of MSI-103 - in particular its azimuthal angle - in the oligomeric assembly, it is now possible to determine and interpret the overall chirality of the proposed toroidal pore.

## Results

The peptide MSI-103 with a ^15^N-label at the backbone amide of Ala-10, plus seven analogs with Ala-d_3_ placed one-by-one in different positions along the sequence, were synthesized and purified. The peptides are listed in Table 1. The parent peptide and all of its analogues have been previously shown by circular dichroism spectroscopy to fold as α-helices in lipid vesicles with the same degree of helicity, and they exhibit essentially the same antibiotic activity against bacteria^16^.

**Table 1:** Synthesized peptides used in this study.

Peptide	Labeled position	Sequence
MSI-103	None	KIAGKIAKIAGKIAKIAGKIA-NH_2_
MSI-103-^15^N	Ala-10	KIAGKIAKI-^**15**^**N-Ala**-GKIAKIAGKIA-NH_2_
MSI-103-A7	Ala-7	KIAGKI-**Ala-d**_**3**_-KIAGKIAKIAGKIA-NH_2_
MSI-103-I9A	Ile-9	KIAGKIAK-**Ala-d**_**3**_-AGKIAKIAGKIA-NH_2_
MSI-103-A10	Ala-10	KIAGKIAKI-**Ala-d**_**3**_-GKIAKIAGKIA-NH_2_
MSI-103-G11A	Gly-11	KIAGKIAKIA-**Ala-d**_**3**_-KIAKIAGKIA-NH_2_
MSI-103-I13A	Ile-13	KIAGKIAKIAGK-**Ala-d**_**3**_-AKIAGKIA-NH_2_
MSI-103-A14	Ala-14	KIAGKIAKIAGKI-**Ala-d**_**3**_-KIAGKIA-NH_2_
MSI-103-A17	Ala-17	KIAGKIAKIAGKIAKI-**Ala-d**_**3**_-GKIA-NH_2_

The highlighted ^15^N-Ala was labeled with ^15^N at the backbone amide, and Ala-d_3_ was labeled with ^2^H at the side chain CH_3_-group.

### ^31^P-NMR

**Figure 2:**
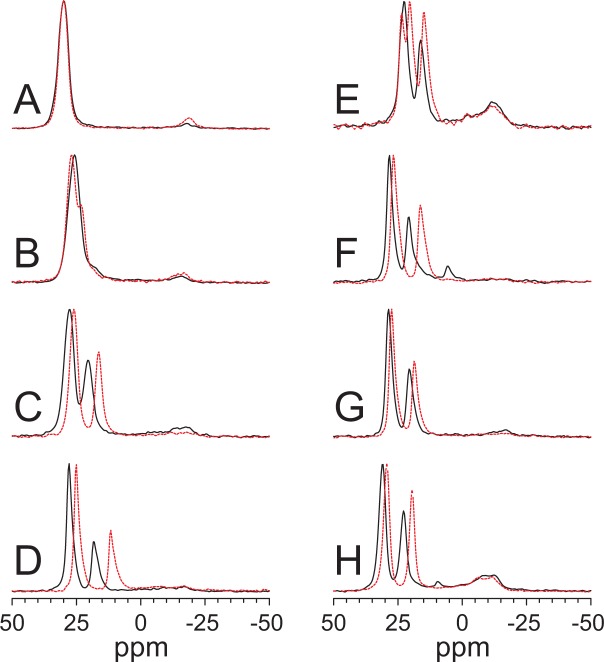
^31^P-NMR spectra of oriented lipid systems without peptide (black solid line) and with ^15^N-MSI-103 at a total P/L = 1/50 (red dashed line). The peak close to 30 ppm is from oriented PC and PG lipids, and the peak at around 20 ppm is from oriented lyso-lipids. (**A**) DMPC; (**B**) DMPC/DMPG (2/1); (**C**) DMPC/lyso-MPC (2/1); (**D**) DMPC/lyso-MPG (2/1); (**E**) DMPC/DMPG/lyso-MPC (1/1/1); (**F**) DMPC/lyso-LPC (2/1); (**G**) DLPC/lyso-MPC (2/1); (**H**) DLPC/lyso-LPC (2/1).

To observe the effect of peptides on the phospholipids and any potential formation of isotropic or disordered lipid phases, ^31^P-NMR was used. The quality of lipid alignment in the samples can be determined from the integrated area of the narrow oriented signals (around 30 ppm for the PC lipids and around 20 ppm for the lyso-lipids) compared to the unoriented powder contribution (with peaks at −10 ppm to −15 ppm). In most cases, the orientation was good, with more than 80% oriented lipids (Fig. 2). Interestingly, the lipid orientation was often found to be better with peptides than without peptides, as seen for example in Fig. 2C,F. In the case of the DMPC system, the position of the narrow oriented ^31^P-NMR peak at 29 ppm did not change upon adding MSI-103 at P/L = 1:50 (Fig. 2 and Table 2), but in all other lipid systems there was an effect. In pure DMPC/DMPG (2/1) bilayers the two lipid peaks (PC and PG) overlapped to give one peak at 26 ppm, while in the presence of the peptide the PC peak shifted to a higher chemical shift and the PG peak in the opposite direction. These shifts indicate a preferred interaction of the cationic MSI-103 with the anionic PG lipids but not with the zwitterionic (uncharged) PC. For PC/lyso-PC (2/1) samples, the PC signal was found at 25 ppm, shifted 4 ppm due to the presence of zwitterionic lyso-MPC. With MSI-103, the DMPC peak was shifted 1–2 ppm, while the lyso-PC signal was shifted 3–5 ppm. This result shows again that the interaction between lyso-PC and peptide is stronger than that between PC and peptide, indicating the possibility that the lyso-lipids are, on average, closer to the peptides. In a sample with DMPC/DMPG/lyso-MPC (1/1/1), the PC signal did not change in the presence of peptide; however, there was a large shift of the PG signal, and the lyso-MPC signal shifted less than for the DMPC/lyso-MPC mixture. This result was expected, since the cationic MSI-103 and the anionic PG lipids should attract each other, under the assumption that PG interacts stronger with the peptide than with lyso-PC. In a DMPC/lyso-MPG sample, there was a very large shift of the lyso-MPG signal, also indicating a strong interaction between the cationic peptide and the anionic lyso-PG lipids.

**Table 2:** Change in ^31^P-NMR chemical shift with and without peptide (P/L = 1/50)^a^.

Lipid system	Chemical shift change (ppm)		
	PC	Lyso-PC	PG/lyso-PG
DMPC	+0.1		
DMPC/DMPG (2/1)	+1.2		−2.4
DMPC/lyso-MPC (2/1)	−1.8	−4.2	—
DMPC/lyso-MPG (2/1)	−3.0	−	−6.6
DMPC/DMPG/lyso-MPC (1/1/1)	+0.1	−1.5	−2.8
DMPC/lyso-LPC (2/1)	−1.7	−4.3	—
DLPC/lyso-MPC (2/1)	−1.2	−2.2	—
DLPC/lyso-LPC (2/1)	−1.4	−3.0	—

^a^Average chemical shift of oriented peaks in samples with peptide subtracted from chemical shift in samples without peptide.

The ^31^P-NMR signals were also found to shift slightly during the long durations of the less sensitive ^2^H- and ^15^N-NMR experiments, presumably due to slight sample dehydration under the high-power rf-irradiation. Therefore, quick ^31^P-NMR measurements were always performed before and after the long NMR experiments on the labeled peptides (which typically ran for 20–24 h). The ^31^P chemical shift was usually found to be increased by 1–2 ppm after these experiments (Table 3), but no significant differences were seen between the PC, PG and lyso-lipid ^31^P-NMR signals, which were all shifted to a similar degree. There was at no time any indication of a change in the lipid morphology, such as phase state or other dehydration-induced effects.

**Table 3:** Change in ^31^P-NMR chemical shift during SSNMR experiments^a^.

Lipid system	Chemical shift change (ppm)		
	PC	Lyso-PC	PG/lyso-PG
DMPC/lyso-MPC (2/1)	1.7	2.2	—
DMPC/lyso-MPG (2/1)	2.2	—	2.5
DMPC/DMPG/lyso-MPC (1/1/1)	0.7	0.8	2.1
DMPC/lyso-LPC (2/1)	1.0	1.4	—
DLPC/lyso-MPC (2/1)	1.3	1.0	—
DLPC/lyso-LPC (2/1)	1.3	1.5	—

^a^Chemical shift of oriented peaks after the experiment subtracted from chemical shift before the experiment in the same sample. Values are averages over seven ^2^H-NMR and one ^15^N-NMR samples. Both ^15^N- and ^2^H-NMR experiments were run typically for 20–24 h.

### ^15^N-NMR

#### Varying DMPC/lyso-MPC ratios

**Figure 3:**
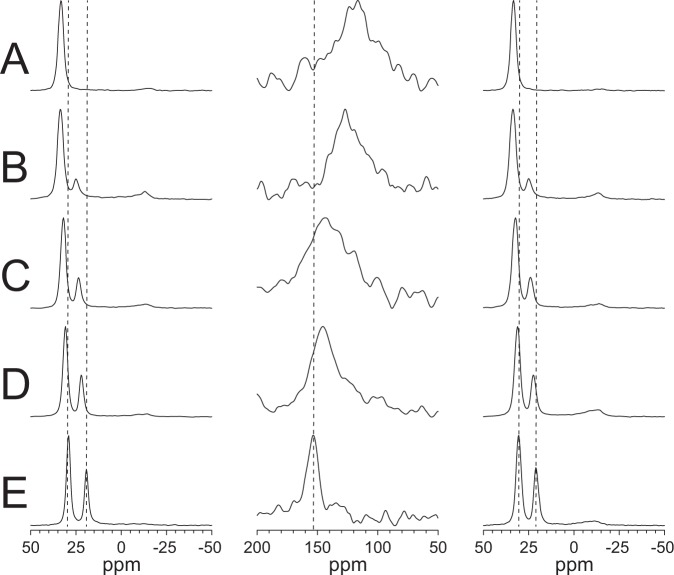
Solid-state NMR spectra of ^15^N-MSI-103 in different lipid systems at P/L = 1/50. The left column shows ^31^P-NMR spectra before the ^15^N-experiment, the middle column shows ^15^N-NMR spectra, and the right column shows ^31^P-NMR spectra after the ^15^N-experiment. (**A**) DMPC only; (**B**) DMPC/lyso-MPC (6/1); (**C**) DMPC/lyso-MPC (4/1); (**D**) DMPC/lyso-MPC (3/1); (**E**) DMPC/lyso-MPC (2/1). Vertical dashed lines are added to the columns to guide the eye.

It has been previously shown that an increase in the positive spontaneous curvature of the lipid system generally favors the insertion of peptides and proteins into membranes^20^. With increasing amounts of lyso-MPC in DMPC bilayers, it can therefore be expected that more peptides should become inserted, or that the peptides should become inserted more deeply into the hydrophobic core. Here, ^15^N-NMR experiments were performed on MSI-103–^15^N in oriented bilayers containing between 0 and 33 mol-% lyso-MPC. Spectra are shown in Fig. 3. The orientation of the α-helical peptide can be estimated from the ^15^N chemical shift; a peak at approximately 90 ppm indicates that the peptide lies essentially flat on the membrane (i.e. parallel to its surface), whereas a peak at approximately 200 ppm indicates that the peptide is in an upright transmembrane orientation^32–35^. Notably, the ^15^N-NMR signals of MSI-103 are seen to be shifted dramatically with increasing lyso-lipid content, reaching characteristic ppm values that are indicative of an inserted orientation. Without lyso-PC, the ^15^N chemical shift was 120 ppm in pure DMPC; in the presence of 14% (mol/mol) lyso-MPC it moved to 128 ppm, at 20–25% to 146 ppm, and at 33% it reached 154 ppm which is indicative of the most inserted peptides. This gradual shift implies that increasing amounts of lyso-MPC enable the peptides to more favorably assemble into a transmembrane pore, either by stabilizing the lifetime of the pores (within the millisecond timescale of the NMR experiment) or by allowing more and more peptides to participate. We also note that the (2/1 = 33 mol-%) sample also gave the narrowest signal, which may be due to a more uniform average orientation of peptides. Therefore, in all further experiments 33% of lyso-lipids were used in the lipid matrix. A chemical shift of 149 ppm has been previously reported in DMPC/lyso-MPC^18^, which is close to the value found here.

#### Different P/L ratios in DMPC with zwitterionic lyso-MPC

**Figure 4:**
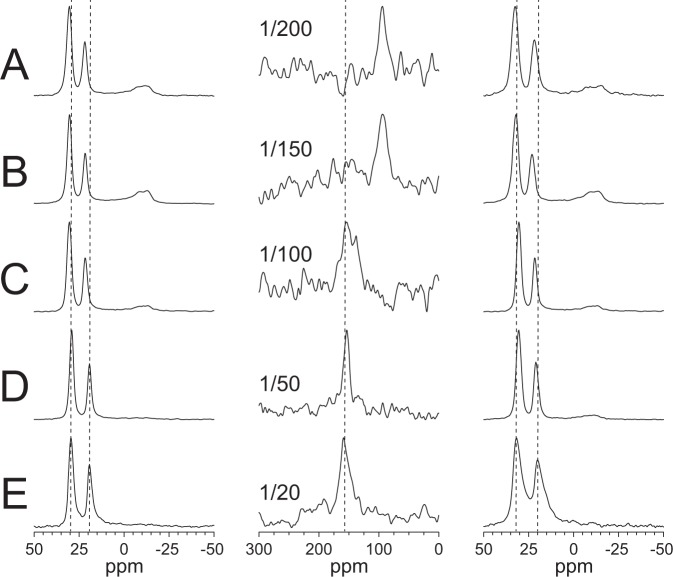
Solid-state NMR spectra of ^15^N-MSI-103 in DMPC/lyso-MPC (2/1) at different P/L. The left column shows ^31^P-NMR spectra before the ^15^N-experiment, the middle column shows ^15^N-NMR spectra, and the right column shows ^31^P-NMR spectra after the ^15^N-experiment. (**A**) P/L = 1/200; (**B**) P/L = 1/150; (**C**) P/L = 1/100; (**D**) P/L = 1/50; (**E**) P/L = 1/20. Vertical dashed lines are added to the columns to guide the eye.

The next question is whether the peptide orientation in lyso-lipid containing membranes changes with peptide concentration, since pore formation is a cooperative process. ^15^N-NMR is not a very sensitive method and therefore not suitable to test very low P/L values; however, it was possible to perform qualitative experiments with ^15^N-MSI-103 in DMPC/lyso-MPC (2/1) on P/L values between 1/20 and 1/200 (Fig. 4). At low peptide concentration (P/L values of 1/200 and 1/150), a chemical shift of approximately 120 ppm was found, which is the same as for MSI-103 in DMPC alone. At a higher P/L of 1/100, the chemical shift increased to 154 ppm, and the same result was found at P/L values of 1/50 and 1/20. These results indicate that MSI-103 is surface-bound at low peptide concentration, but at and above a concentration threshold of P/L = 1/100 the helices become inserted. We note that the NMR peaks were somewhat broader at a lower peptide concentration, possibly due to noise or to a broader underlying distribution of tilt angles. For all following experiments, we therefore used P/L = 1/50, which gave narrow signals of peptides in the inserted state.

#### Different P/L ratios in DMPC with anionic lyso-MPG

**Figure 5:**
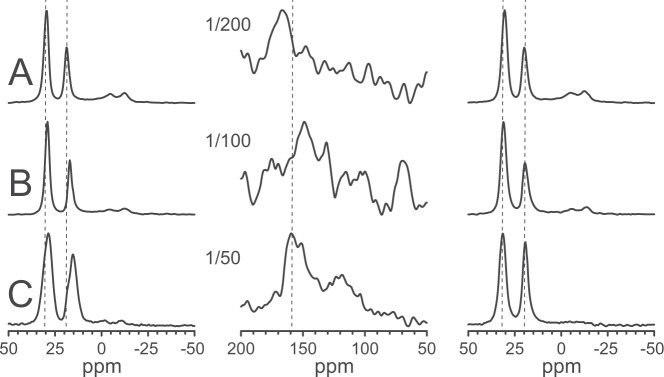
Solid-state NMR spectra of ^15^N-MSI-103 in DMPC/lyso-MPG (2/1) at different molar peptide-to-lipid (P/L) ratios, (giving only a poor signal-to-noise at low peptide concentration). The left column shows ^31^P-NMR spectra before the ^15^N-experiment, the middle column shows ^15^N-NMR spectra, and the right column shows ^31^P-NMR spectra after the ^15^N-experiment. (**A**) P/L = 1/200; (**B**) P/L = 1/100; (**C**) P/L = 1/20. Vertical dashed lines are added to the columns to guide the eye.

There is an electrostatic attraction between cationic peptides and anionic lipids, and we have demonstrated above that lyso-lipids are accumulated at the peptidic pores. Therefore, it is likely that anionic lyso-MPG would interact even stronger with the peptide than zwitterionic lyso-MPC. This expectation to promote pore formation further is supported by the ^15^N-NMR spectra of ^15^N-MSI-103 in DMPC/lyso-MPG (2:1) at P/L = 1/200, 1/100, and 1/20 shown in Fig. 5. In this case, despite the low spectral quality, we see that the peptide peak reached 170 ppm already at the lowest peptide concentration of 1/200, indicating that peptides were already inserted in the membrane to form pores.

#### Other lipid systems

**Figure 6:**
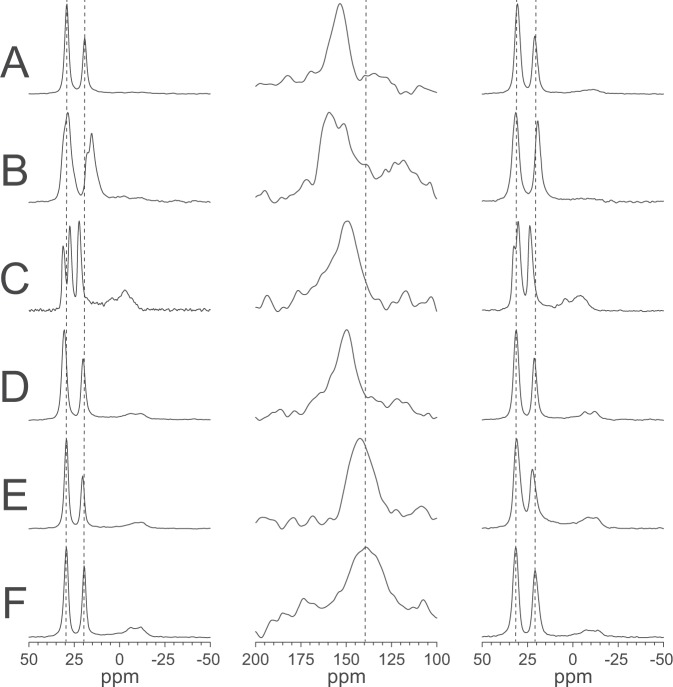
Solid-state NMR spectra of ^15^N-MSI-103 in different lipid systems at P/L = 1/50. The left column shows ^31^P-NMR spectra before the ^15^N-experiment, the middle column shows ^15^N-NMR spectra, and the right column shows ^31^P-NMR spectra after the ^15^N-experiment. (**A**) DMPC/lyso-MPC (2/1); (**B**) DMPC/lyso-MPG (2/1); (**C**) DMPC/DMPG/lyso-MPC (1/1/1); (**D**) DMPC/lyso-LPC (2/1); (**E**) DLPC/lyso-MPC (2/1); (**F**) DLPC/lyso-LPC (2/1). Vertical dashed lines are added to the columns to guide the eye.

It was found earlier that MSI-103 in DMPC/lyso-MPC (2/1) gives a ^15^N-NMR signal at approximately 150 ppm, indicating an essentially upright orientation at P/L = 1/50 in which the peptide spans the membrane^18^. Peptides of different lengths were used in that study, and the helix tilt angle was found to increase with increasing peptide length, such that the peptide length was always matched to the membrane hydrophobic thickness^18^. If this hydrophobic matching applies also in the presence of lyso-lipids, then the tilt angle of MSI-103 would be expected to adapt itself to membranes of different thickness. Therefore, ^15^N-NMR was measured for MSI-103 in lipids with shorter chains. DLPC (di-12:0-PC) was used in place of DMPC (di-14:0-PC), and lyso-LPC was used in place of lyso-MPC. Different combinations of the lipid matrix and the lyso-lipid were examined, in order to check whether the acyl chain length of either the PC component or the lyso-PC component was more important. A 2/1 ratio of PC over lyso-PC and a P/L = 1/50 was kept throughout. The ^15^N-NMR spectra are shown in Fig. 6, and the ^15^N chemical shift values of the peaks are given in Table 4. In DMPC/lyso-MPC (2/1), a chemical shift of 153 ppm was observed. In DMPC/lyso-LPC (2/1), the shift changed only slightly to 150 ppm, which may indicate a minor increase in tilt. In DLPC/lyso-MPC (2/1), there was a larger effect, as the chemical shift decreased to 142 ppm, indicating a significantly more tilted state. In DLPC/lyso-LPC (2/1), the chemical shift was the lowest (139 ppm), indicating the largest tilt of the peptide helix, as expected for a mismatch-dependent effect. Clearly, the response of the peptide was most pronounced when the 66% DMPC matrix was replaced by DLPC, whereas changing the 33% lyso-MPC to lyso-LPC had a much smaller effect on the tilt.

**Table 4:** ^15^N-NMR chemical shifts of ^15^N-labeled MSI-103 in the studies lipid systems.

Lipid system	Chemical shift (ppm)
DMPC/lyso-MPC (2/1)	153.4
DMPC/lyso-MPG (2/1)	159.3
DMPC/DMPG/lyso-MPC (1/1/1)	149.0
DMPC/lyso-LPC (2/1)	149.6
DLPC/lyso-MPC (2/1)	142.4
DLPC/lyso-LPC (2/1)	139.0

Additionally, the effect of charge in the lipid matrix was studied. In one experiment, anionic DMPG was included to give a 1/1 DMPC/DMPG mixture, and in another experiment the lyso-MPC was replaced by anionic lyso-MPG. In both samples, 1/3 of the total lipids were negatively charged, while maintaining the same acyl chain composition. Electrostatic interactions of DMPG and lyso-MPG with the cationic MSI-103 are expected, just as they were seen by ^31^P-NMR. It is plausible that – according to the generally accepted toroidal wormhole model of a pore – lyso-lipids should be more favorably accommodated within the highly curved walls of the pore, which consist of both, lipids and peptide molecules^36^. In that case, the cylindrical DMPG and the cone-shaped lyso-MPG lipids should draw MSI-103 more towards the surface-bound state or more into the inserted state, respectively. Indeed, in DMPC/DMPG/lyso-MPC (1/1/1), MSI-103 displayed a slightly smaller ^15^N chemical shift of 149 ppm and hence peptides seems to be less inserted than in DMPC/lyso-MPC (1/1) with 153 ppm, while in DMPC/lyso-MPG (1/1) the chemical shift increased to 159 ppm, indicative of the strongest insertion and most pronounced pore forming effect (Fig. 6). The effect of DMPG is small, but fully supports the idea that lyso-lipids are enriched within the curved walls of the pore compared to the lamellar regions of the matrix. Note that in this very dynamical liquid crystalline system the observed ^15^N signal represents a time average over the entire ensemble of peptides. Therefore, a larger chemical shift value simply means that the average orientation of the MSI-103 helix is more upright in DMPC/lyso-MPG, suggesting that a higher proportion of peptides is present in a pore state and/or it remains there for a longer duration of time.

### ^2^H-NMR

#### 3D structure in different lipid systems

**Figure 7:**
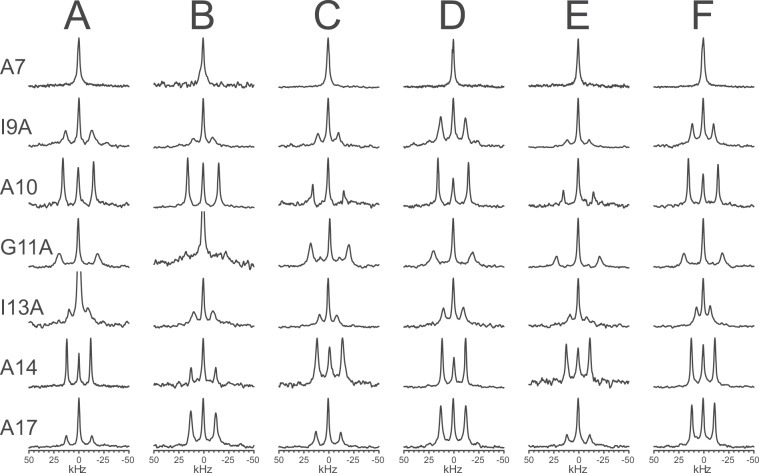
^2^H-NMR spectra of MSI-103 labeled at different positions (as marked for each row) in different lipid systems at P/L = 1/50. (**A**) DMPC/lyso-MPC (2/1); (**B**) DMPC/lyso-MPG (2/1); (**C**) DMPC/DMPG/lyso-MPC (1/1/1); (**D**) DMPC/lyso-LPC (2/1); (**E**) DLPC/lyso-MPC (2/1); (**F**) DLPC/lyso-LPC (2/1).

To obtain the full set of orientational information on MSI-103, the peptide was selectively labeled with Ala-d_3_ at seven different positions for comprehensive ^2^H-NMR structure analysis. For several relevant systems with different lipid compositions, oriented samples were prepared with all seven ^2^H-labeled peptides, always using a total P/L of 1/50. The resulting ^2^H-NMR spectra are shown in Fig. 7. In Figs. S1–S6 in the Supporting Information, ^31^P-NMR spectra before and after each ^2^H-NMR experiment are shown for each lipid system, to demonstrate that the bilayers were well-oriented and that the samples did not dry out during the experiment. In each ^2^H-spectrum, there was a central peak originating from traces of deuterium in the water (even though samples were prepared using deuterium-depleted water) and possibly originating from small amounts of detached peptide that is undergoing fast isotropic motion.

The characteristic feature of these ^2^H-NMR spectra is the doublet, symmetrically distributed around the central peak, which represents the labeled peptide bound to the membrane. The quadrupole splitting depends on the average angle θ between the C-CD_3_ bond and the external magnetic field, being proportional to ½(3cos^2^θ − 1). It can be noted that the splitting has an intrinsic sign, but this cannot be obtained from the spectra, and at a specific angle called the magic angle (≈54.7°) the splitting is zero. From the set of quadrupole splittings of all seven labels, and based on the assumption of a regular α-helical structure, the orientation of the peptide backbone in the membrane can be calculated^37,38^.

In all lipid systems (Fig. 7), splittings from the bound peptide were visible in all spectra except for those from Ala-d_3_ at position 7. Only in DMPC/lyso-LPC, there was a small splitting of 1.2 kHz seen for position 7, but in all other cases the dominant central line showed no resolved splitting. This means that this particular C-CD_3_ bond is aligned close to the magic angle, hence the splitting was set to zero in the data analysis. All quadrupolar splittings from the different labeled peptides in the different lipid systems are listed in Table 5. It can be noted that the splittings from any specific position are usually very similar to within ±2 kHz for all lipid systems, with just a few exceptions. This observation indicates that the peptide orientation must also be quite similar in these different environments. A detailed analysis was performed by fitting the ^2^H-NMR splittings to so-called helical waves, and the resulting best-fit parameters are listed in Table 6.

**Table 5:** ^2^H-NMR splittings (in kHz) of Ala-d_3_ labeled MSI-103 analogs in the studied lipid systems.

Lipid	Ala-d_3_ labeled position						
	7	9	10	11	13	14	17
DMPC/lyso-MPC (2/1)	0	26.3	30.7	38.3	18.7	23.9	25.5
DMPC/lyso-MPG (2/1)	0	19.2	31.0	39.8	19.1	24.8	24.9
DMPC/DMPG/lyso-MPC (1/1/1)	0	20.6	30.9	38.2	17.1	22.8	24.8
DMPC/lyso-LPC (2/1)	1.2	24.7	31.6	39.1	19.9	23.6	25.0
DLPC/lyso-MPC (2/1)	0	21.9	30.3	42.9	16.5	23.7	22.7
DLPC/lyso-LPC (2/1)	0	21.3	29.7	38.4	14.0	23.6	22.4

**Table 6:** Best-fit orientation of MSI-103 in different lipid systems determined from ^2^H-NMR data.

Lipid system	Orientation parameters				
	τ (°)	ρ (°)	σ_τ_ (°)	σ_ρ_ (°)	RMSD (kHz)
DMPC/lyso-MPC (2/1)	151	89	25	1	2.2
DMPC/lyso-MPG (2/1)	151	90	26	0	3.9
DMPC/DMPG/lyso-MPC (1/1/1)	152	91	27	1	3.0
DMPC/lyso-LPC (2/1)	150	88	24	1	2.3
DLPC/lyso-MPC (2/1)	146	92	27	0	2.4
DLPC/lyso-LPC (2/1)	151	91	28	0	2.5

**Figure 8:**
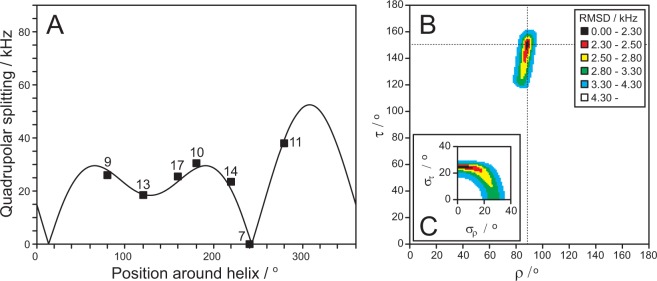
^2^H-NMR data analysis to determine the orientation of MSI-103 in DMPC/lyso-MPC (2/1) lipid bilayers at P/L = 1/50. (**A**) The experimental data points fitted to helical curves with all data points projected to one turn around the helix. Numbers indicate the labeled position corresponding to each data point. (**B**) The RMSD for the best fit is a function of τ and ρ angles and is color-coded at each point. The best fit values are indicated by the intersection of the dotted lines. (**C**) The RMSD for the best fit as a function of σ_τ_ and σ_ρ_. The color coding is the same in (**B**) and **(C**).

For MSI-103 in DMPC/lyso-MPC, the quality of the fit of the calculated helical wave to the experimental data points is illustrated in Fig. 8A. The corresponding τ-ρ plot, indicating the RMSD for different peptide orientations, is given in Fig. 8B. The tilt angle (τ), defined between the helix axis and the bilayer normal, was found to have a best-fit value at 151°. This means that the helix is tilted by only 30° (by symmetry 150° + 30° = 180°) away from the membrane normal, indicating a more or less upright transmembrane orientation of the peptide. The rotation angle (ρ) of 89° means that the charged Lys residues are located on that side of the helix which faces towards the direction of tilt. The dynamical analysis (Fig. 8C) gave a moderate fluctuation of the tilt angle (σ_τ_) of ±25° and essentially no fluctuation of the rotation angle (σ_ρ_ ≈ 0). The root mean square deviation (RMSD) between experimental and calculated splittings was quite low with a value of 2.2 kHz, which is similar to the values found for MSI-103 in other lipid systems using previous ^2^H-NMR data analysis^19^.

In the other lyso-lipid systems tested, almost identical orientation and dynamics were found as in DMPC/lyso-MPC, as seen in Table 6. Fitting curves and RMSD plots are shown in Figs. S7–S11 in the Supporting Information. The tilt angle was almost the same in all six studied systems, namely 149° ± 3°; the rotation angle was 90° ± 2°, the σ_τ_ was 26° ± 2°, and the σ_ρ_ was 0–1°. These are only small differences, within the estimated error of the method, so no significant changes were found between these different lipid systems which contained 33 mol% lyso-lipids. Apparently the same type of pore structure is formed, irrespective of the lipid, at least for these tested systems.

## Discussion

In this study, we used solid-state ^31^P-, ^15^N-, and ^2^H-NMR to characterize the orientation of MSI-103 (also known as KIA21) in membranes containing lyso-lipids, which have been recently demonstrated to support the formation of stable pores. Previously, we had studied the orientation of MSI-103 in many other lipid systems using ^2^H- and ^19^F-NMR^16,19^, where the orientation was found to be dominated by the spontaneous curvature of the lipids. Namely, in lipids with a negative spontaneous curvature such as DOPC, POPC or POPE/POPG, the peptide lay flat on the membrane surface with a tilt angle of approximately 90°. In lipids such as DMPC with a small positive spontaneous curvature, a distinctly different state was observed with a tilt angle of approximately 120°, but this state was not compatible with a membrane-spanning alignment either^16,19^. The same kind of behavior has been described for several other amphipathic α-helical peptides^15,20^, so it was proposed that in general, a more positive spontaneous curvature favors the insertion of peptides into the membrane^20^. Thus, by using lyso-lipids with a high positive spontaneous curvature, it should be possible to induce and stabilize a genuine membrane-spanning orientation of peptides such as MSI-103. This state, compatible with a transmembrane pore, could indeed be demonstrated recently in a systematic study of model peptides with different lengths, derived from the repetitive sequence of MSI-103^18^, where MSI-103 was also included under the name of KIA21. The observed upright transmembrane orientation was proposed to correspond to peptides lining a membrane-spanning water-filled pore. Here, using several lyso-lipid containing lipid systems, it has been finally possible to characterize in detail the underlying structure and deduce the chirality and interpret the supramolecular architecture of such a pore formed by MSI-103.

First, the membrane morphology and its response to the peptide was examined by ^31^P-NMR, as the ^31^P signal originates from the phosphate groups of the phospholipid matrix (including lyso-lipids). Figure 3 shows that the ^31^P signal from well-oriented DMPC shifts towards the isotropic position (at 0 ppm) when more lyso-MPC is added, and so does the lyso-MPC peak. For DMPC/lyso-MPC (2/1), Fig. 2 shows that the lipid peaks shift correspondingly when peptide is added (P/L = 1/50). This can be attributed to a change in the orientation of the ^31^P tensor in the lipid head group relative to the membrane normal, and/or to a change in dynamics in the presence of peptide. The peptide has a positive net charge, and it can be noted that the shift of lipid peaks is greatest for anionic DMPG and lyso-MPG lipids (Table 2), indicating that electrostatic attraction is responsible for at least part of the effect. Electrostatic attraction is also important in the first stage where an unordered peptide in aqueous phase is attracted towards the bilayer and folds into an α-helix. Since the shift is larger for uncharged lyso-PC than for PC, this result was also an indication of a stronger interaction of peptides with the lyso-PC lipids than with the PC matrix.

An opposite effect was observed over the time course of experiments. After measuring the sample in an NMR probe at 308 K for approximately 24 h, the oriented lipid peaks shifted to somewhat higher ppm values (Table 3). This effect is attributed to progressive dehydration of the sample (reducing dynamics), as it was also time dependent, with larger shifts for samples staying longer in the NMR probe. Furthermore, upon rehydrating the samples we obtained very similar chemical shifts as in the fresh ones before the first experiment. We note that this effect, in which lipid ^31^P-NMR peaks were shifted during the experiment, was not observed in samples with only PC or PG lipids; only when lyso-lipids were also present, then the PC and PG peaks shifted.

The insertion of peptides into membranes has been proposed to be favored in bilayers with positive spontaneous curvature, e.g. when they contain lyso-lipids. Using ^15^N-NMR, we indeed observed here that MSI-103 assumed a more and more upright helix orientation with increasing content of lyso-lipids, as seen in Fig. 3. In pure DMPC at P/L = 1/50, the ^15^N-NMR signal from MSI-103 was found at 120 ppm, which had been previously shown by ^2^H- and ^19^F-NMR to correspond to an obliquely tilted helix with a time-averaged angle of 125°^16,19^. This means that the helix axis is tilted far off the bilayer normal, hence it is presumably embedded obliquely within the amphiphilic region at the membrane surface. For symmetry reasons, this tilt angle is equivalent to a value of 55° (as 125° + 55° = 180°), hence it is most likely that two helixes are arranged as an antiparallel X-shaped dimer (with a wide cross-over angle of 2 × 55° relative to the membrane normal) to stabilize each other’s oblique tilt angle.

In the presence of 14 mol-% lyso-MPC in DMPC (molar ratio of 6/1), there was a slight shift of the ^15^N-NMR signal to 128 ppm, which indicated a somewhat more tilted orientation. At 20 mol-% lyso-MPC, the signal shifted further to 146 ppm, and at 25 mol-% and 33 mol-% it moved to 150 ppm. The latter value is consistent with a membrane-spanning helix that has a tilt angle of only 30°, in full agreement with the recently reported value of 150° (as 30° + 150° = 180°)^18^. From these results, we can conclude that approximately 20 mol-% lyso-lipids were sufficient to induce an essentially transmembrane orientation of MSI-103. Higher concentrations of lyso-lipids led to a slight additional shift towards 154 ppm, and also gave much narrower peptide signals. This seems to be due to a more homogeneous population with a unique orientation; in lower lyso-lipid contents, due to an insufficient amount of lyso-lipids, most of the peptides are still obliquely suspended on the surface or are only temporarily inserted in a transmembrane pore. In DMPC/lyso-MPC 2/1, however, essentially all peptides are inserted, and they all have the same well-defined orientation.

A fundamental question regarding the transmembrane orientation of peptides in postulated pores in lyso-lipid systems is whether such pores are formed by the lipids themselves, so that peptides are just partitioned into pre-existing pores, or whether the pores are only formed due to the presence of the peptides. One possible way of testing this is to vary the peptide concentration. If peptides are preferentially located in existing pores, a transmembrane orientation should also be observed at very low peptide concentrations; however, if the peptides themselves induce the pores, peptide-peptide interactions are necessary, hence a certain minimum peptide concentration should be needed for pore formation. We used a high DMPC/lyso-MPC molar ratio of 2/1 and incorporated increasing concentrations of MSI-103 from P/L = 1/200 to 1/20. As seen in Fig. 4A,B, at P/L = 1/200 and 1/150, the peptide signal was found at 95 ppm, which indicated that peptides were lying flat on the membrane surface, with τ ≈ 90°. (It should be noted that this is different from the 120 ppm signal in DMPC at 1/50 in Fig. 3A, which indicates an obliquely tilted peptide.) In the concentration series at 1/100, the peak jumped to 155 ppm, to remain there also at 1/50 and 1/20 (Fig. 4C–E). From this observation of a distinct threshold concentration we can conclude that the pores were not preformed, but they are indeed a result of peptide-peptide interactions. At low peptide concentrations, such interactions are not prominent enough, therefore pores are only formed at P/L = 1/100 and higher (in DMPC/lyso-MPC, 2/1).

Notably, when we replaced the zwitterionic lyso-MPC with anionic lyso-MPG, there was enhanced electrostatic attraction between the cationic peptides and the lyso-lipids, so in this case the pores had already formed at the much lower peptide concentration of 1/200 (Fig. 5A). This finding indicates that the lyso-lipids must be accumulated in the pores, such that pore formation gets further enhanced when the peptides and lyso-lipids are attracted to one another. This is a strong indication that the pores formed are of the toroidal wormhole type^39^, with both, peptides and lipids, lining the water-filled interior of the pore.

Under conditions where pores are formed and peptides are inserted into the membrane, it had been previously found using ^15^N-NMR that the tilt angle changes with peptide length in a mismatch-dependent manner^18^. Longer peptides were more tilted, so the peptide length was clearly able to adapt to the membrane thickness. If longer peptides are more tilted in membranes of a constant thickness, it can also be expected that peptides of a constant length will be more tilted in thinner membranes. To test this hypothesis, MSI-103 was compared in DMPC/lyso-MPC and in thinner DLPC/lyso-LPC membranes, as well as in mixtures of DMPC/lyso-LPC and DLPC/lyso-MPC. As seen in the ^15^N-NMR data (Fig. 6 and Table 4), the peptides were indeed more upright in the thicker membranes, and the effect of changing acyl chains was larger when applied to the PC matrix than to the lyso-PC component. This behavior was expected, because each PC lipid has two acyl chains, whereas each lyso-PC lipid has only one, and because a PC/lyso-PC ratio of 2/1 was used.

### Model of peptide-lipid interactions and pore formation

**Figure 9:**
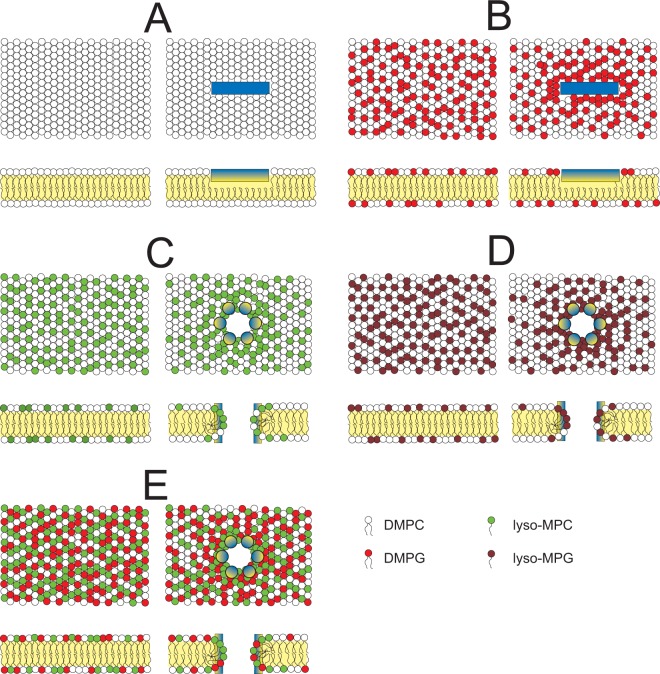
Models of peptide-lipid interactions and pores in the membrane, seen from the top and side views of the lipid bilayer. The peptide arrangement in pores is shown only schematically here, but more details are given in Fig. 10. The left part of each panel shows the bilayer without peptide, the right part with MSI-103 (blue/yellow cylinder). Lipids are color-coded as shown. (**A**) Bilayer of only DMPC. (**B**) DMPC/DMPG (2:1) bilayer. (**C**) Pore formed in a DMPC/lyso-MPC (2:1) bilayer with transmembrane peptides; the pore is enriched in lyso-PC compared to the bulk membrane. (**D**) Pore formed in a DMPC/lyso-MPG (2:1) bilayer with transmembrane peptides. Here, the pore is further enriched with lyso-PG. (**E**) Pore formed in DMPC/DMPG/lyso-MPC (1:1:1) bilayer with transmembrane peptides.

We can now use the set of SSNMR data to propose a model of the peptide-lipid interactions and describe the behavior of both, peptides and lipids. In a membrane composed of pure DMPC, MSI-103 was bound to the surface and was oriented mostly in a flat S-state (Fig. 9A). There was hardly any change in the ^31^P chemical shift of DMPC upon binding of MSI-103, suggesting no strong interaction (Table 2) nor any significant membrane perturbation (Fig. 9A). In DMPC/DMPG (3/1), the lipids seem to be well mixed in the absence of any peptide (Fig. 9B), as they both have essentially the same ^31^P chemical shift of 25.6 ppm, which is much lower than PC alone giving an oriented peak at 30 ppm (Fig. 2). When MSI-103 was added, the PG ^31^P chemical shift decreased to 23 ppm, while it increased to 27 ppm for PC. This observation indicates that anionic DMPG was attracted to the cationic peptide, hence the annular membrane patch around the peptide was enriched in PG, while further away the matrix got relatively depleted in its anionic component (Fig. 9B). Such an effect has been described as lipid clustering, i.e. cationic peptides and proteins are known to concentrate negatively charged lipids around themselves, and indeed MSI-103 shows a very strong tendency to cluster anionic lipids^13^. This ability of the peptide combined with the observation of an inserted state of the helix in the membrane suggests the formation of a toroidal wormhole. When peptides and lyso-lipids were present in sufficient amounts, stable oligomeric structures were formed, in which the peptides were aligned in an upright transmembrane orientation (i.e. with the helix axis almost parallel to the membrane normal), implying that the amphiphilic peptides form the scaffold that defines the pore (Fig. 9C). Between the peptide molecules, there are also lipids found to be present in the pore, especially an enrichment of lyso-lipids, hence this assembly is clearly of the toroidal wormhole type. The pore is further stabilized by electrostatic interactions between peptides and anionic lyso-lipids (here: lyso-MPG, as opposed to lyso-MPC), because in this lipid composition the pores were already formed at distinctly lower concentrations of peptide and lyso-lipid (Fig. 9D). In a mixture of zwitterionic cylindrical DMPC, anionic cylindrical DMPG and anionic cone-shaped lyso-MPC (Fig. 9E), there will be a certain competition between DMPG and lyso-PG, allowing some enrichment of charged DMPG around the peptides, but this does not exclude lyso-MPC from getting close to and especially inbetween the upright peptide helices, so a pore is still formed as seen from the ^15^N-NMR data (Fig. 6C).

### Pore models derived from the orientational geometries of the peptide helices

To determine the orientation of MSI-103 more accurately, we performed comprehensive ^2^H-NMR experiments on peptides that were selectively labeled with Ala-d_3_. Such a single substitution does not influence the secondary structure or activity of the peptide^16^. While the analysis of a single ^15^N label reveals only an approximate tilt angle of the peptide in the membrane, several Ala-d_3_ labels yield not only a more accurate tilt angle (τ), but also the azimuthal rotation angle (ρ) as well as dynamical parameters^37,38,40,41^. This method was used to study MSI-103 in several lipid systems containing 33 mol-% lyso-lipids at P/L = 1/50. In all six systems, the splittings were very similar (Table 5), hence the orientation was found to be very similar in all cases (Table 6 and Figs. 8, S7–S11): τ = 149° ± 3°, ρ = 90° ± 2°, σ_τ_ = 26° ± 2°, and σ_ρ_ = 0°–1°. The observed range of orientational angles lies within the estimated error of the method. This result is seemingly different from the ^15^N-NMR results, where the chemical shift of the single peak varied a bit between the lipid systems (Table 4). However, in the ^2^H-NMR analysis, seven data points were used to determine the orientation, which makes this method more reliable and accurate than ^15^N-NMR, where there is only a single (sometimes quite broad) peak from which the tilt angle can be estimated. Even though the individual ^2^H-NMR splittings for a specific labeled position changed up to 7 kHz between the different lipid systems, the overall fit using all splittings gave a tilt angle variation of just ±3° for the different lipids. Thus, relying on only one label, as in the ^15^N-NMR experiments, might give uncertain results, whereas the use of more labels should give a more reliable result. It should also be mentioned that the ^15^N-NMR chemical shift is very sensitive in this range of tilt angles and is close to the magic angle, so a minor deviation in the manually alignment of the oriented sample can lead to a significant change in the observed chemical shift. Overall, we conclude that the differences in the orientation of MSI-103 in these PC/lyso-PC lipid systems are within the error margin of the method and that the orientation is essentially the same in all cases.

Interestingly, also the dynamical behavior of the inserted peptide helices is very similar in all cases tested here, and quite characteristic with σ_τ_ = 26° ± 2°, and σ_ρ_ = 0°–1°. In a previous study of the orientation of MSI-103 in various different lipid systems, some rather different dynamics were obtained in the absence of lyso-lipids, i.e. under conditions where the peptide was aligned more or less flat on the membrane surface^19^. There, σ_τ_ was close to 0°, whereas σ_ρ_ was around 15°–25°. In that previous study, also one sample of POPC/lyso-OPC (1:1) had been included, and indeed in this singular system we found σ_τ_ = 19°, and σ_ρ_ = 14°. It thus seems that in the presence of lyso-lipids, there is generally a larger fluctuation of the peptide tilt angle, but a smaller fluctuation of the rotation angle. This dynamical behavior fits well with the toroidal pore hypothesis. Peptides in a pore can readily slide along the curved membrane surface and change their tilt angle relative to the overall sample normal, whereas peptides on a flat membrane surface must displace hydrophobic lipid acyl chains in order to be able to change their tilt significantly. At the same time, when the upright peptides in a pore form dimeric building blocks (see below) or are otherwise intertwined in tight architectures, this would make it harder to change their azimuthal rotation angle, which may explain the low value of σ_ρ_. It might be interesting to find out whether a barrel-stave pore (as proposed, e.g., for the much more hydrophobic alamethicin peptaibol), would exhibit rather different dynamical parameters compared to a toroidal wormhole pore, given that a smooth variation in tilt angle should be less feasible in a barrel-stave pore.

**Figure 10:**
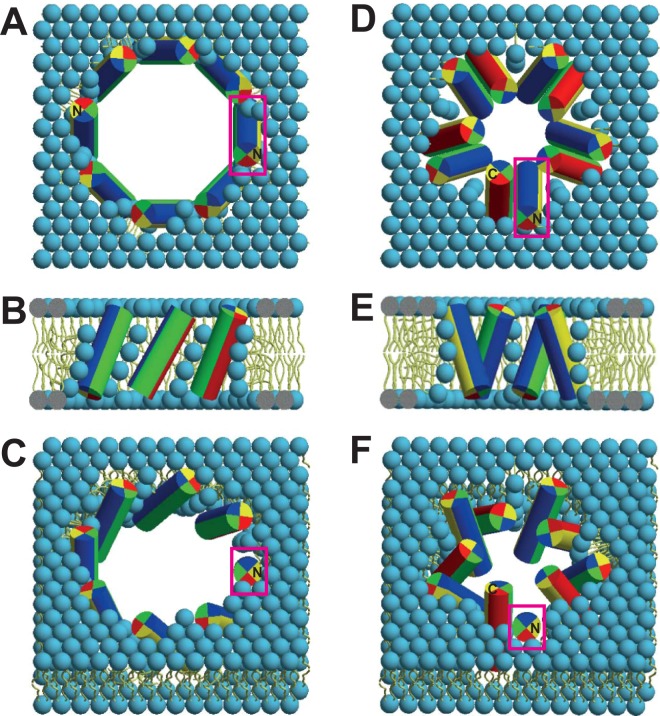
Assembly of the MSI-103 helices into a toroidal wormhole pore, according to their tilt angle (τ = 150°) and azimuthal rotation (ρ = 90°) obtained here by ^2^H-NMR. All peptides and lipids are colored as in Fig. 1 (Lys: blue, Ile: yellow, Ala: red, Gly: green). (**A**) Top, (**B**) side and (**C**) tilted views of the assembly of an (unknown) number of monomers, giving a bundle with a left-handed overall chirality. In this scenario, the Gly-rich faces point into the center of the water-filled pore, while the Lys residues (blue) are positioned sideways. (**D**) Top, (**E**) side and (**F**) tilted views of another plausible model, using the same tilt and azimuthal angles obtained from ^2^H-NMR, but based on the formation of antiparallel dimers. Here, the MSI-103 dimers themselves have a left-handed supercoiled twist. The helix-helix contacts involve the Gly-rich face (green face of the cylinders), allowing at the same time all of the charged Lys residues (blue) on the dimer to point into the water-filled center of the pore. The peptide orientation is the same in both models, as is best seen by comparing the highlighted peptides (in red boxes). In the monomeric model, all peptides have a parallel orientation, with the N-terminus pointing up; whereas in the dimeric model, the peptides have an antiparallel orientation as dimers, with alternatingly N- and C-terminus pointing up. Both models are consistent with the NMR data and are plausible in terms of peptide-lipid interactions, hence further structural approaches are needed to discriminate these two possibilities.

With the azimuthal angle found here for MSI-103, ρ = 90° (and considering the definition of this angle^16^), we obtain a distinct peptide orientation in the toroidal pore that is illustrated in Fig. 10, even though the number of monomers comprising the pore is not accessible from our data. In the first model, Fig. 10A–C suggests that the helices should be arranged with a left-handed sense of chirality. It seems thermodynamically unfavorable, however, that the pore should be lined by the Gly-rich face of the helices, rather than by Lys residues. This arrangement, fixed now by ρ = 90°, is not compatible with either of the initial expectations illustrated in Fig. 1B,C (where the pore is lined by Lys residues). Alternatively, Fig. 10D–F shows a more plausible model of a toroidal pore, which has been constructed by assuming the peptide to assemble as dimers as the basic building block. In this model, two MSI-103 molecules form an antiparallel dimer with a left-handed supercoiled twist, held together by close contacts via the Gly-rich faces (marked in green in Figs. 1 and 10) as has been proposed in the literature for several peptides^42^, including PGLa, the parent peptide of MSI-103^43^. With ρ = 90°, all Lys residues are located on one side of the dimer, which are perfectly positioned now to point into the water-filled pore, as expected. The other sides of the dimer are hydrophobic and point into the hydrophobic part of the membrane. The fact that only a single resonance is observed for MSI-103, implying a unique orientation of the peptide in the inserted state, is fully consistent with an antiparallel dimer, because a parallel dimer arranged in such a way should give rise to two NMR signals. Furthermore, an antiparallel arrangement is energetically favored by the strong intrinsic helix dipole of the folded peptide molecule.

There is some indirect indication in the literature that MSI-103 indeed has a tendency to form dimers, and it makes sense that this should occur via the Gly-rich face, as in the well-known helix-helix interaction motif GxxxG^42,44^. In our earlier solid-state NMR structure analyses of both MSI-103 and of its natural parent PGLa, we had observed a stepwise change in the helix tilt angle when the peptide concentration in the membrane was gradually increased^16,19^. At very low peptide concentration in DMPC, the MSI-103 helix was found to lie essentially flat on the membrane surface in a so-called S-state with τ ≈ 101°^16^ (98° for PGLa^41^), just as has been seen here by ^15^N-NMR. Remarkably, under conditions that did not support pore formation, increasing peptide concentration caused MSI-103 to re-align into a discrete obliquely tilted T-state with τ ≈ 128° (126° for PGLa^41^). Similarly, in the presence of lyso-lipids we have observed here a discrete re-alignment of MSI-103 into yet another state, called the inserted I-state with τ ≈ 150°^16^ (160° for PGLa in the presence of its synergistic partner magainin 2^45^). The fact that the interconversion between the S-state and the T-state is not gradual, but occurs at a threshold concentration, suggests that peptide-peptide interactions are involved in stabilizing the T-state, just as we have seen it here for the I-state corresponding to the toroidal pore. The T-state is not tilted strongly enough to span the hydrophobic membrane, but we can state that this putative surface-bound state is likely to be an antiparallel dimer, because only a single set of NMR resonances is observed – just as for the membrane-spanning I-state described in the present study. So far, the only other example where the 3D pore architecture of peptides from the antimicrobial magainin family has been resolved by solid-state NMR, concerns the 1:1 complex of PGLa and MAG2^46,47^. These two peptides assemble into a stable pore even without lyso-lipids. The transmembrane portion was found to be made up entirely of PGLa dimers, which are stabilized by surface-anchored magainin helices^43^. The role of the latter presumably serves to impose a highly positive local curvature in the membrane.

The fact that peptide tilt is modulated by hydrophobic mismatch, as also seen in our previous ^15^N-NMR study^18^, may be interpreted to support the monomer model of Fig. 10A–C rather than the dimeric building blocks of Fig. 10D–F. That is because (classical) dimers with extensive contacts between the two monomers might be rather constrained at the helix-helix interface, unwilling to adopt their tilt angle to the membrane thickness. Here, however, it was found that the tilt angle can become very large, up to 135° (helix axis tilted 45° from the bilayer normal), as a function of mismatch^18^. This observation would support the monomer model, in which all hydrophobic residues can readily avoid any contact with water, which would be harder to reconcile in a constrained dimer model. However, as pointed out above, the interface of MSI-103 is not a classical GxxxG motif, as it less compact and may actually be more flexible to tolerate a variety of helix-helix crossing angles. Furthermore, we have recently found that the replacement of Gly-11 in MSI-103 by a large and bulky CF_3_-Phg amino acid, leads to a strong reduction in the antimicrobial membranolytic activity^16^. This finding indicates that Gly-11 plays an important role in the mechanism of action of MSI-103, hence the possibility of dimeric antiparallel transmembrane helices with an adjustable crossing-angle cannot be ruled out.

In summary, the solid-state NMR data is compatible with a pore formed either by monomers or by antiparallel dimers, and there are pros and cons for both models, which cannot be discriminated from any accessible data so far.

### Proposed mechanism of action

Regarding the mechanism of pore formation during biological action, we propose the following model: MSI-103 is electrostatically attracted by and will bind in an S-state to the outer membrane of the bacterial cell that is being attacked. Provided that the local peptide concentration is sufficiently high, the peptide will tend to assemble probably as an antiparallel dimer, but remain on the bilayer surface in an obliquely tilted T-state. That is because natural bacterial membranes possess a high content of phosphatidyl-ethanolamine, which imposes a spontaneous negative curvature. An increasing accumulation of surface-bound peptides (S-state and T-state) in the outer monolayer of the membrane, however, will change the lateral pressure profile of the membrane and turn the spontaneous curvature of the outer monolayer into a positive curvature. The peptides will respond cooperatively by inserting more deeply to form the membrane-spanning I-state, which corresponds to the toroidal wormhole pore. The open pore is expected to only be transiently stable in a natural bacterial membrane, namely as long as the peptide concentration on the outer and inner monolayers has not equilibrated. As soon as a sufficient number of peptides has slipped through the toroidal wormhole and equilibrated themselves on the inner monolayer in a T- or S-state, the prevailing spontaneous negative curvature of the bacterial lipids will presumably not support a long-lived pore. Therefore, it is not surprising that previous biophysical attempts based on equilibrated samples (such as NMR/ESR, circular dichroism, diffraction, etc.) have failed to characterize a stable transmembrane pore of an antimicrobial peptide such as MSI-103, when incorporated into natural or bacterial model membranes. Only the trick of incorporating a considerably high proportion of lyso-lipids with an intrinsic positive curvature allowed us to carry out this comprehensive structural analysis.

## Materials and methods

### Materials

The lipids 1,2-dilauroyl-*sn*-glycero-3-phosphatidylcholine (DLPC), 1,2-dimyristoyl-*sn*-glycero-3-phosphatidylcholine (DMPC), 1,2-dimyristoyl-*sn*-glycero-3-phosphatidylglycerol (DMPG), 1-myristoyl-2-hydroxy-*sn*-glycero-3-phosphatidylcholine (lyso-MPC), 1-myristoyl-2-hydroxy-*sn*-glycero-3-phosphatidylglycerol (lyso-MPG), and 1-lauroyl-2-hydroxy-*sn*-glycero-3-phosphatidylcholine (lyso-LPC) were purchased from Avanti Polar Lipids (Alabaster, AL, USA).

### Peptide synthesis

Peptides were synthesized using standard solid phase Fmoc protocols, as described previously^16^.

### Solid-state NMR

#### NMR sample preparation

Macroscopically oriented NMR samples were prepared by co-dissolving typically 1 mg of peptide and appropriate amounts of lipids to get the wanted peptide-to-lipid molar ratio (P/L). Peptides were first dissolved in 20 µL MilliQ-water to which was added 80 µL of methanol. Lipids were dissolved in chloroform/methanol (1/1 v/v) or chloroform/methanol/ MilliQ-water (65/35/8 v/v/v), and the lipid solution added to the peptide solution, giving a clear mixed solution. This solution was spread onto 20–25 thin glass plates of dimensions 9 mm × 7.5 mm × 0.08 mm (Marienfeld Laboratory Glassware, Lauda-Königshofen, Germany). The plates were dried in air for 1 h, followed by drying under vacuum overnight. The dried plates were stacked and hydrated at 48 °C for 18–24 h in a chamber with 96% relative humidity. Hydrated samples were wrapped in parafilm and plastic foil to protect from drying during the NMR measurements.

#### NMR experimental details

All NMR measurements were performed at 308 K on a Bruker Avance spectrometer (Bruker Biospin, Karlsruhe, Germany) with a 500 or 600 MHz ^1^H Larmor frequency. The macroscopically oriented samples were placed in a flat-coil probe with the lipid bilayer normal aligned parallel to the magnetic field. ^31^P-NMR was done to examine the lipid orientation, using a Hahn echo sequence with ^1^H decoupling using a previously published phase cycling^48^. The ^31^P chemical shift was referenced using the water signal in a ^1^H single-pulse experiment run immediately before or after the ^31^P-NMR experiment on the same sample in the same probe. This water signal at 308 K was set to 4.65 ppm^49^, and the reference frequency of ^31^P was calculated from the reference frequency of ^1^H according to the gyromagnetic ratios^50^. ^2^H-NMR experiments were performed as described previously^51^ using a quadrupole echo sequence^52^ with a 90° pulse of 4.5 μs, an echo delay of 70 μs, a 100 ms relaxation delay time, a 500 kHz spectral width, and 2048 data points. Between 100,000 and 1,000,000 scans were collected. ^1^H-^15^N cross polarization experiments were performed using a double-tuned home-built probe with a low-E flat-coil resonator (3 mm × 9 mm cross section), using a CP-MOIST pulse sequence^53^ employing a ^1^H and ^15^N radiofrequency field strength of 65 kHz during cross polarization, and 36 kHz ^1^H SPINAL16 decoupling^54^ during acquisition. The acquisition time was 10 ms, and the recycle time 3 s. A mixing time of 500 ms was used, and between 10,000 and 30,000 scans were accumulated. The ^15^N chemical shift was referenced using the signal of an ammonium sulfate dry powder sample set to 26.8 ppm.

#### NMR data analysis

^2^H-NMR data was analyzed as previously described^51^. In short, the orientation of a helical peptide in the membrane can be defined by two angles, the helix tilt angle τ and the azimuthal rotation angle ρ. τ is defined as the angle between the long axis of the helix, pointing from the N- to the C-terminus, and the membrane normal. A τ of 0° would mean that the peptide is oriented with the C-terminus on the top of the membrane, whereas 180° would have the N-terminus on the top. 90° tilt means the peptide is lying flat on the membrane surface. The azimuthal rotation angle ρ defines the rotation of the peptide around its long axis. The ρ angle is defined to be 0° when the vector in a plane perpendicular to the helix axis from the helix axis to the Cα of Lys-12 is parallel to the plane of the membrane^19,40^. The dynamical behavior of a helix is characterized in terms of a rigid body wobble of these two parameters, quantified by the standard deviations σ_τ_ and σ_ρ_ of the corresponding Gaussian distribution functions. Using ^2^H-NMR data from seven individually Ala-d_3_ labeled positions, the helix orientation was calculated from RMSD fits and quadrupolar wave plots, as described previously^19,37,38,40,41,51,55^.

## Supplementary information

Supplementary Information.

## References

[1] Brogden KA (2005). Antimicrobial peptides: pore formers or metabolic inhibitors in bacteria?. Nat. Rev. Microbiol..

[2] Pinheiro da Silva F, Machado MC (2012). Antimicrobial peptides: clinical relevance and therapeutic implications. Peptides.

[3] Wimley WC, Hristova K (2011). Antimicrobial peptides: successes, challenges and unanswered questions. J. Membr. Biol..

[4] Boman HG (1991). Antibacterial peptides: key components needed in immunity. Cell.

[5] Lee MT, Sun TL, Hung WC, Huang HW (2013). Process of inducing pores in membranes by melittin. Proc. Natl. Acad. Sci. USA.

[6] Lee CC, Sun Y, Qian S, Huang HW (2011). Transmembrane pores formed by human antimicrobial peptide LL-37. Biophys. J..

[7] Qian S, Wang WC, Yang L, Huang HW (2008). Structure of transmembrane pore induced by Bax-derived peptide: evidence for lipidic pores. Proc. Natl. Acad. Sci. USA.

[8] Qian S, Wang WC, Yang L, Huang HW (2008). Structure of the alamethicin pore reconstructed by x-ray diffraction analysis. Biophys. J..

[9] Yang L, Harroun TA, Weiss TM, Ding L, Huang HW (2001). Barrel-stave model or toroidal model? A case study on melittin pores. Biophys. J..

[10] Maloy WL, Kari UP (1995). Structure-activity studies on magainins and other host-defense peptides. Biopolymers.

[11] Soravia E, Martini G, Zasloff M (1988). Antimicrobial properties of peptides from *Xenopus* granular gland secretions. FEBS Lett..

[12] Strandberg E (2007). Influence of C-terminal amidation on the antimicrobial and hemolytic activities of cationic α-helical peptides. Pure Appl. Chem..

[13] Wadhwani P (2012). Membrane-active peptides and the clustering of anionic lipids. Biophys. J..

[14] Grau-Campistany A (2015). Hydrophobic mismatch demonstrated for membranolytic peptides, and their use as molecular rulers to measure bilayer thickness in native cells. Sci. Rep..

[15] Strandberg E, Zerweck J, Wadhwani P, Ulrich AS (2013). Synergistic insertion of antimicrobial magainin-family peptides in membranes depends on the lipid spontaneous curvature. Biophys. J..

[16] Strandberg E (2008). Solid state NMR analysis comparing the designer-made antibiotic MSI-103 with its parent peptide PGLa in lipid bilayers. Biochemistry.

[17] Bürck J (2008). Conformation and membrane orientation of amphiphilic helical peptides by oriented circular dichroism. Biophys. J..

[18] Grau-Campistany A, Strandberg E, Wadhwani P, Rabanal F, Ulrich AS (2016). Extending the hydrophobic mismatch concept to amphiphilic membranolytic peptides. J. Phys. Chem. Lett..

[19] Strandberg E, Tiltak D, Ehni S, Wadhwani P, Ulrich AS (2012). Lipid shape is a key factor for membrane interactions of amphipathic helical peptides. Biochim. Biophys. Acta.

[20] Strandberg E, Ulrich AS (2015). AMPs and OMPs: is the folding and bilayer insertion of β-stranded outer membrane proteins governed by the same biophysical principles as for α-helical antimicrobial peptides?. Biochim. Biophys. Acta.

[21] Gagnon MC (2017). Influence of the length and charge on the activity of α-helical amphipathic antimicrobial peptides. Biochemistry.

[22] Strandberg E, Ulrich AS (2004). NMR methods for studying membrane-active antimicrobial peptides. Concepts Magn. Reson. A.

[23] Wadhwani, P. *et al*. Using fluorinated amino acids for structure analysis of membrane-active peptides by solid-state ^19^F-NMR in *Current Fluoroorganic Chemistry: New Synthetic Directions, Technologies, Materials, and Biological Applications ACS Symposium Series 949* (eds. Soloshonok, V. A. *et al*.) 431–446 (American Chemical Society, 2007).

[24] Ulrich AS (2005). Solid state ^19^F-NMR methods for studying biomembranes. Progr. Nucl. Magn. Reson. Spect..

[25] Strandberg, E. & Ulrich, A. S. Solid-state NMR for studying peptide structures and peptide-lipid interactions in membranes in *Modern Magnetic* Resonance (ed. Webb, G.) (Springer, 2017).

[26] Strandberg, E. & Ulrich, A. S. Solid-state ^19^F-NMR analysis of peptides in oriented biomembranes in *Modern Magnetic* Resonance (ed. Webb, G.) (Springer, 2017).

[27] Bechinger B, Salnikov ES (2012). The membrane interactions of antimicrobial peptides revealed by solid-state NMR spectroscopy. Chem. Phys. Lipids.

[28] Gehman, J. D. & Separovic, F. Solid-state NMR of membrane-active proteins and peptides in *Modern Magnetic Resonance* Vol. 1 (ed. Webb, G. A.) 305–311 (Springer, 2006).

[29] Sani MA, Separovic F (2015). Progression of NMR studies of membrane-active peptides from lipid bilayers to live cells. J. Magn. Reson..

[30] Bhattacharjya S, Ramamoorthy A (2009). Multifunctional host defense peptides: functional and mechanistic insights from NMR structures of potent antimicrobial peptides. FEBS J..

[31] Liu MH, Zhang L, Wang TY (2015). Supramolecular chirality in self-assembled systems. Chem. Rev..

[32] Bechinger B, Gierasch LM, Montal M, Zasloff M, Opella SJ (1996). Orientations of helical peptides in membrane bilayers by solid state NMR spectroscopy. Solid State Nucl. Magn. Reson..

[33] Bechinger B (1991). Orientations of amphipathic helical peptides in membrane bilayers determined by solid-state NMR spectroscopy. J. Biomol. NMR.

[34] Henzler Wildman KA, Lee DK, Ramamoorthy A (2003). Mechanism of lipid bilayer disruption by the human antimicrobial peptide, LL-37. Biochemistry.

[35] Cross TA, Opella SJ (1994). Solid-state NMR structural studies of peptides and proteins in membranes. Curr. Opin. Struct. Biol..

[36] Hallock KJ, Lee DK, Ramamoorthy A (2003). MSI-78, an analogue of the magainin antimicrobial peptides, disrupts lipid bilayer structure via positive curvature strain. Biophys. J..

[37] Strandberg E (2004). Tilt angles of transmembrane model peptides in oriented and non-oriented lipid bilayers as determined by ^2^H solid state NMR. Biophys. J..

[38] Van der Wel PCA, Strandberg E, Killian JA, Koeppe RE (2002). Geometry and intrinsic tilt of a tryptophan-anchored transmembrane α-helix determined by ^2^H NMR. Biophys. J..

[39] Ludtke SJ (1996). Membrane pores induced by magainin. Biochemistry.

[40] Strandberg E, Esteban-Martín S, Salgado J, Ulrich AS (2009). Orientation and dynamics of peptides in membranes calculated from ^2^H-NMR data. Biophys. J..

[41] Strandberg E, Wadhwani P, Tremouilhac P, Dürr UHN, Ulrich AS (2006). Solid-state NMR analysis of the PGLa peptide orientation in DMPC bilayers: structural fidelity of ^2^H-labels versus high sensitivity of ^19^F-NMR. Biophys. J..

[42] Russ WP, Engelman DM (2000). The GxxxG motif: a framework for transmembrane helix-helix association. J. Mol. Biol..

[43] Zerweck, J. *et al*. Molecular mechanism of synergy between the antimicrobial peptides PGLa and magainin 2. *Sci. Rep*. **7** (2017). 10.1038/s41598-017-12599-7 PMC5640672 29030606

[44] Senes A, Engel DE, DeGrado WF (2004). Folding of helical membrane proteins: the role of polar, GxxxG-like and proline motifs. Curr. Opin. Struct. Biol..

[45] Strandberg E, Tremouilhac P, Wadhwani P, Ulrich AS (2009). Synergistic transmembrane insertion of the heterodimeric PGLa/magainin 2 complex studied by solid-state NMR. Biochim. Biophys. Acta.

[46] Tremouilhac P, Strandberg E, Wadhwani P, Ulrich AS (2006). Synergistic transmembrane alignment of the antimicrobial heterodimer PGLa/magainin. J. Biol. Chem..

[47] Salnikov ES, Bechinger B (2011). Lipid-controlled peptide topology and interactions in bilayers: structural insights into the synergistic enhancement of the antimicrobial activities of PGLa and magainin 2. Biophys. J..

[48] Rance M, Byrd RA (1983). Obtaining high-fidelity spin-1/2 powder spectra in anisotropic media - phase-cycled Hahn echo spectroscopy. J. Magn. Reson..

[49] Gottlieb HE, Kotlyar V, Nudelman A (1997). NMR chemical shifts of common laboratory solvents as trace impurities. J. Org. Chem..

[50] Markley JL (1998). Recommendations for the presentation of NMR structures of proteins and nucleic acids - (IUPAC Recommendations 1998). Pure Appl. Chem..

[51] Strandberg E (2018). Helix fraying and lipid-dependent structure of a short amphipathic membrane-bound peptide revealed by solid-state NMR. J. Phys. Chem. B.

[52] Davis JH, Jeffrey KR, Bloom M, Valic MI, Higgs TP (1976). Quadrupolar echo deuteron magnetic resonance spectroscopy in ordered hydrocarbon chains. Chem. Phys. Lett..

[53] Levitt MH, Suter D, Ernst RR (1986). Spin dynamics and thermodynamics in solid-state NMR cross polarization. J. Chem. Phys..

[54] Fung BM, Khitrin AK, Ermolaev K (2000). An improved broadband decoupling sequence for liquid crystals and solids. J. Magn. Reson..

[55] Glaser RW (2005). Concentration-dependent realignment of the antimicrobial peptide PGLa in lipid membranes observed by solid-state ^19^F-NMR. Biophys. J..

