# Supplementary material for: Chiral supramolecular architecture of stable transmembrane pores formed by an α-helical antibiotic peptide in the presence of lyso-lipids
Source: Sci Rep. 2020 Mar 13;10:4710. doi: 10.1038/s41598-020-61526-w (PMC7070102; doi:10.1038/s41598-020-61526-w)
Supplement: Supplementary file 1 — Supplementary Information. [file 41598_2020_61526_MOESM1_ESM.pdf]

Supporting Information

Chiral supramolecular architecture of stable transmembrane pores formed by an α-helical antibiotic peptide in the presence of lyso-lipids

Erik Strandberg[a], David Bentz[b], Parvesh Wadhwani[a], and Anne S. Ulrich[a,b,]*

[a] Karlsruhe Institute of Technology (KIT), Institute of Biological Interfaces (IBG-2), POB 3640, 76021 Karlsruhe, Germany; [b] KIT, Institute of Organic Chemistry, Fritz-Haber-Weg 6, 76131 Karlsruhe, Germany.

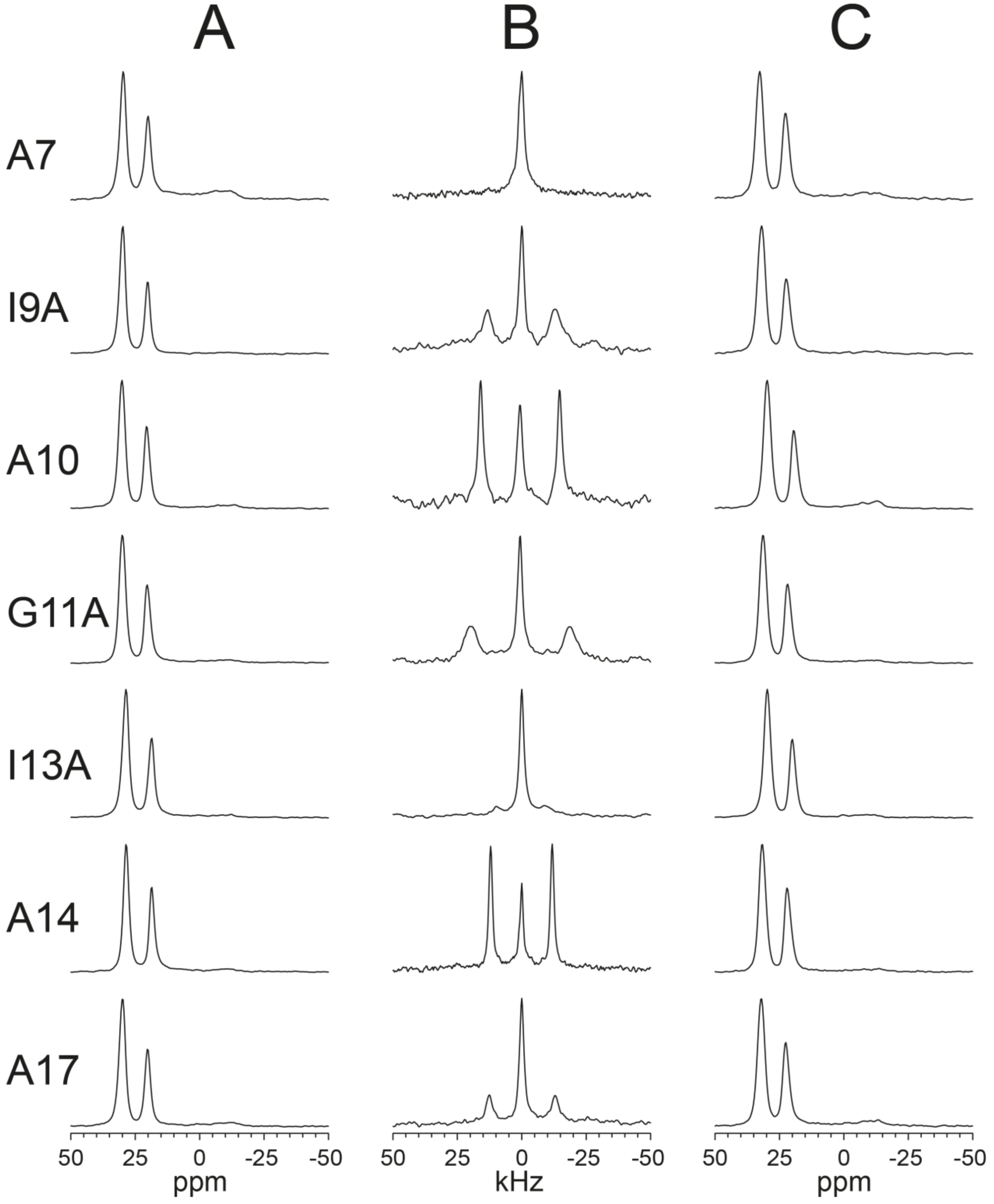

Figure S1. ^{31}P- and ^{2}H-NMR spectra of Ala-d_3 labeled MSI-103 in DMPC/lyso-MPC (2/1). The labeled position is given to the left of each row. (A) ^{31}P-NMR spectra before ^{2}H-NMR experiment. (B) ^{2}H-NMR spectra. (C) ^{31}P-NMR spectra after ^{2}H-NMR experiment.

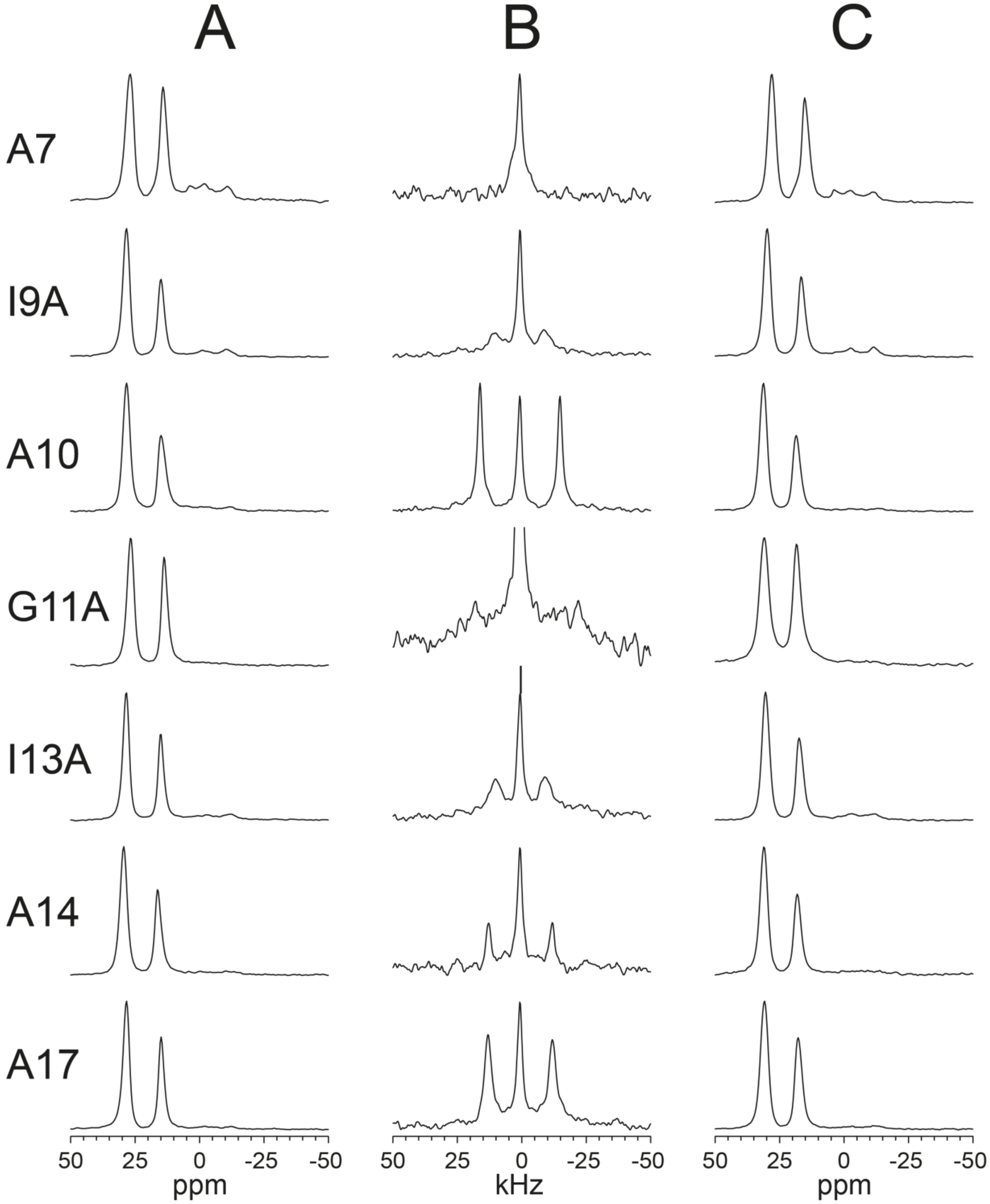

Figure S2. ^{31}P- and ^{2}H-NMR spectra of Ala-d_3 labeled MSI-103 in DMPC/lyso-MPG (2/1). (A) ^{31}P-NMR spectra before ^{2}H-NMR experiment. (B) ^{2}H-NMR spectra. (C) ^{31}P-NMR spectra after ^{2}H-NMR experiment.

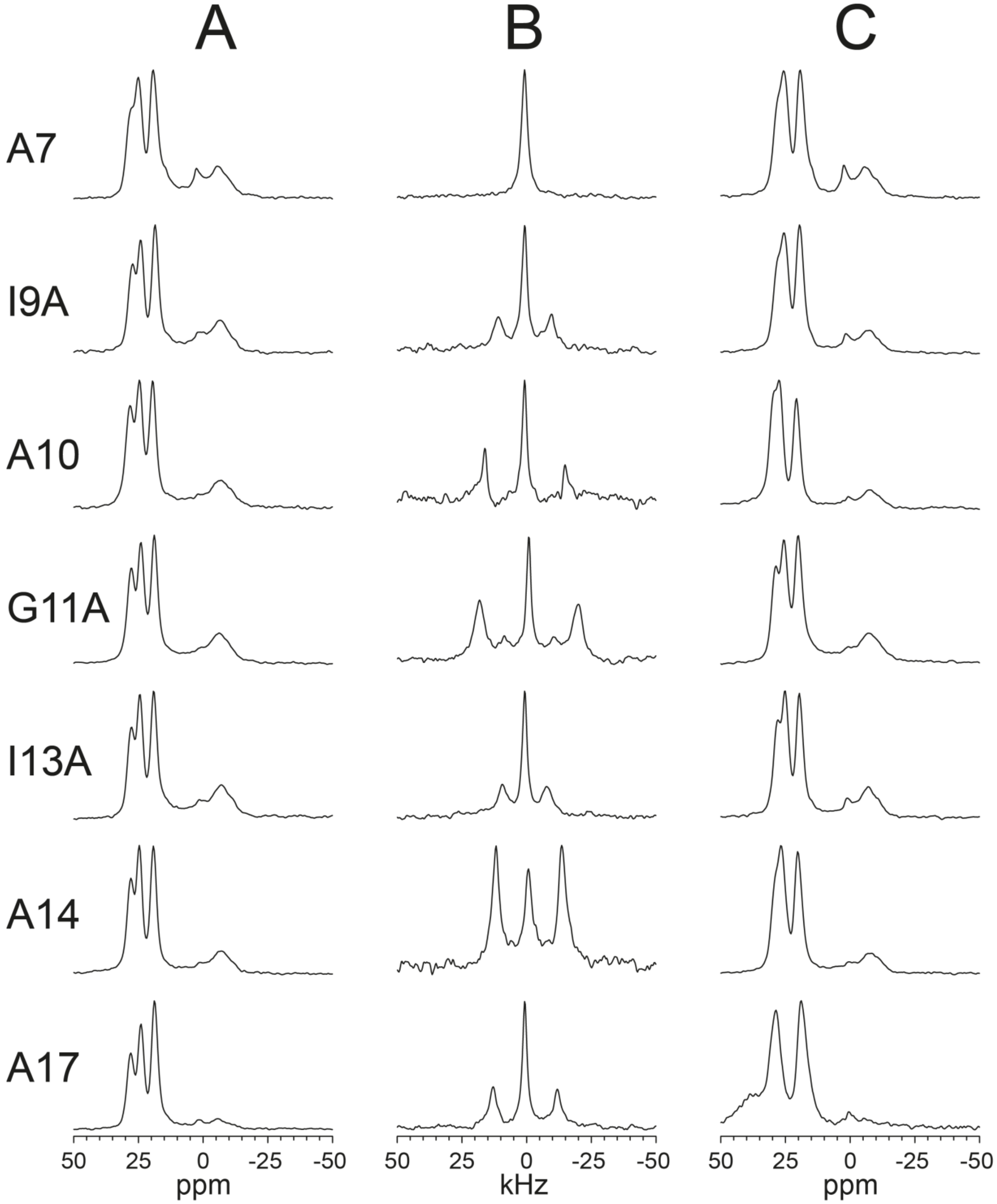

Figure S3. ^{31}P- and ^{2}H-NMR spectra of Ala-d_3 labeled MSI-103 in DMPC/DMPC/lyso-MPC (1/1/1). (A) ^{31}P-NMR spectra before ^{2}H-NMR experiment. (B) ^{2}H-NMR spectra. (C) ^{31}P-NMR spectra after ^{2}H-NMR experiment.

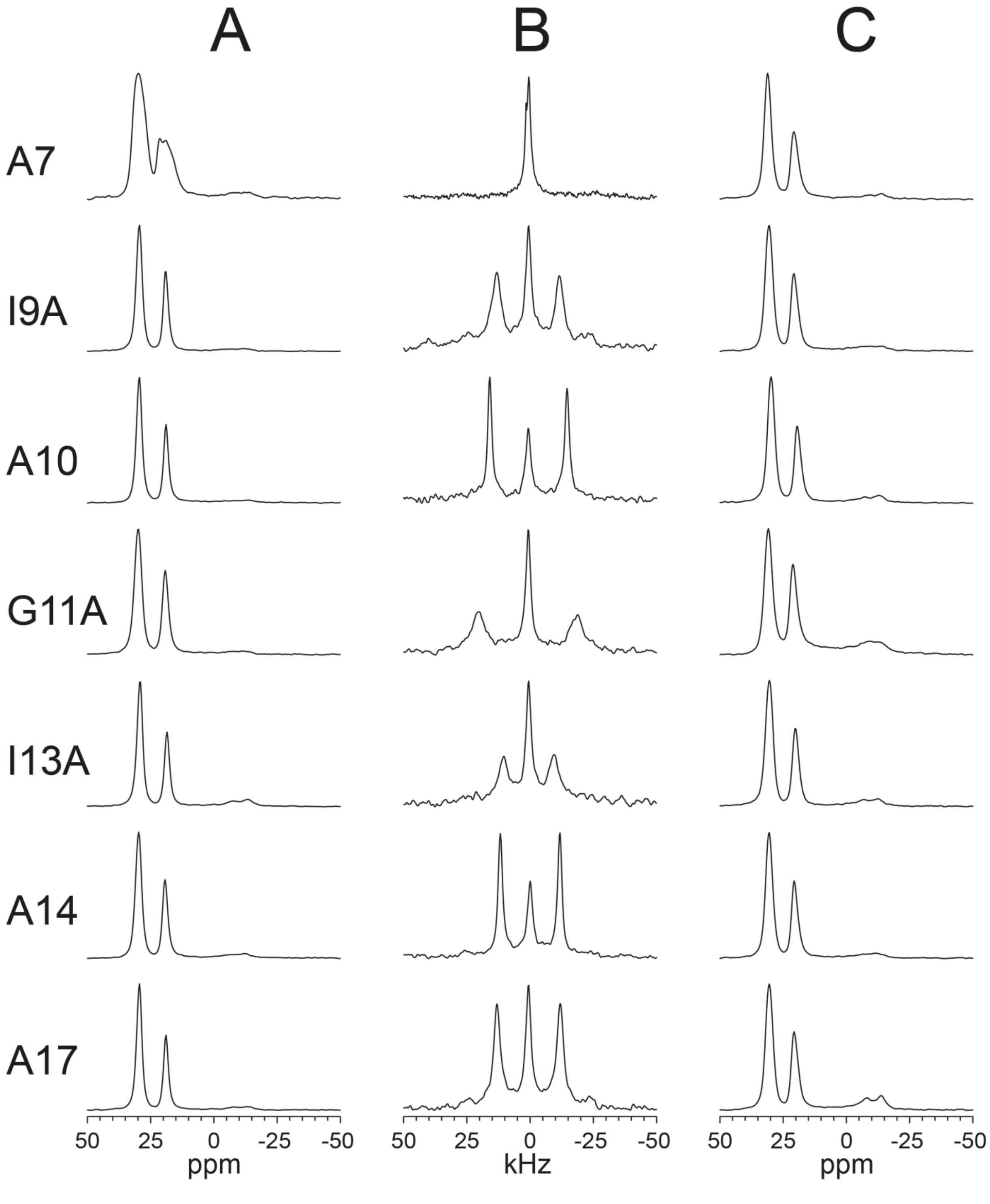

Figure S4. ^{31}P- and ^{2}H-NMR spectra of Ala-d_3 labeled MSI-103 in DMPC/lyso-LPC (2/1). (A) ^{31}P-NMR spectra before ^{2}H-NMR experiment. (B) ^{2}H-NMR spectra. (C) ^{31}P-NMR spectra after ^{2}H-NMR experiment.

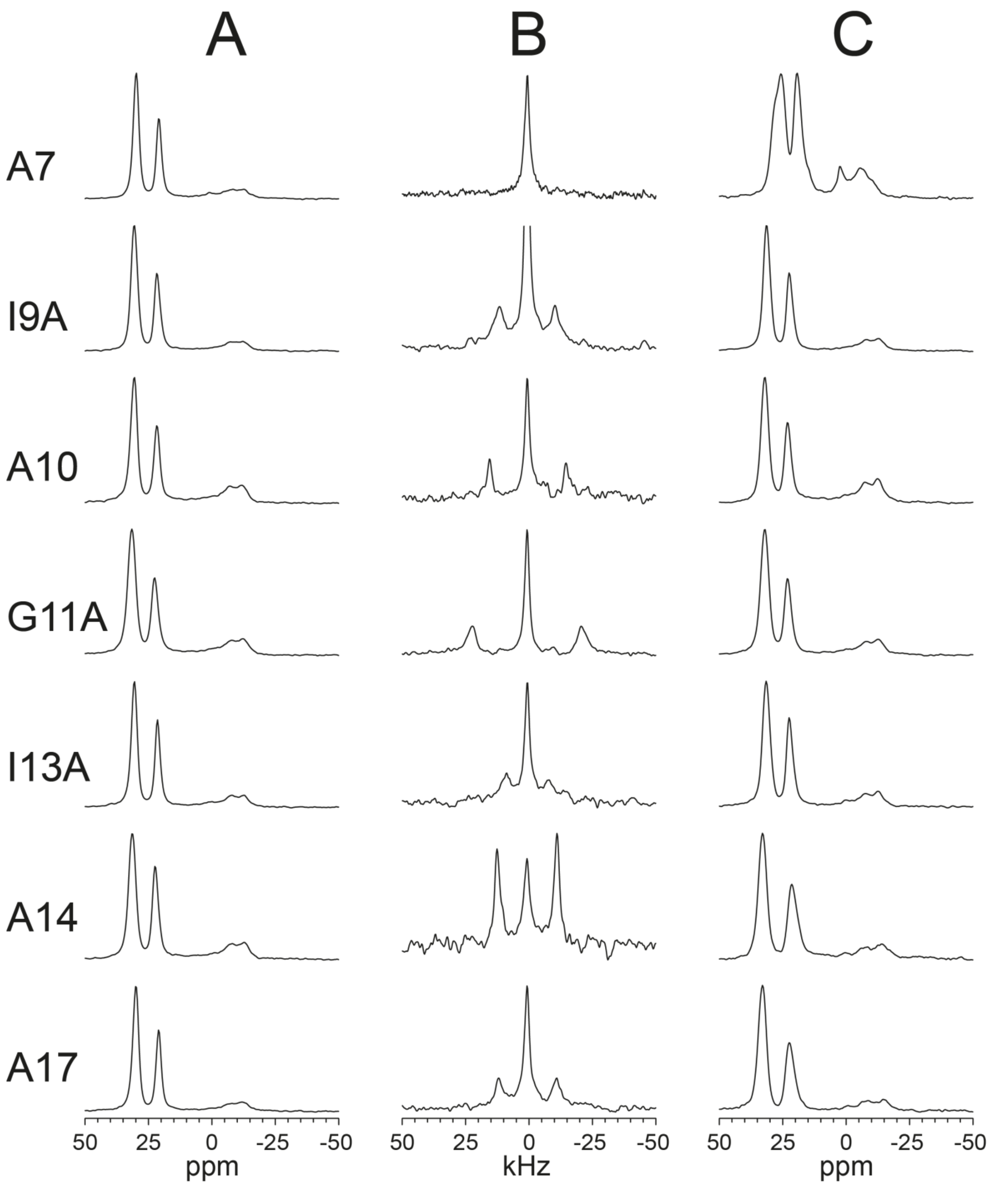

Figure S5. ^{31}P- and ^{2}H-NMR spectra of Ala-d_3 labeled MSI-103 in DLPC/lyso-MPC (2/1). (A) ^{31}P-NMR spectra before ^{2}H-NMR experiment. (B) ^{2}H-NMR spectra. (C) ^{31}P-NMR spectra after ^{2}H-NMR experiment.

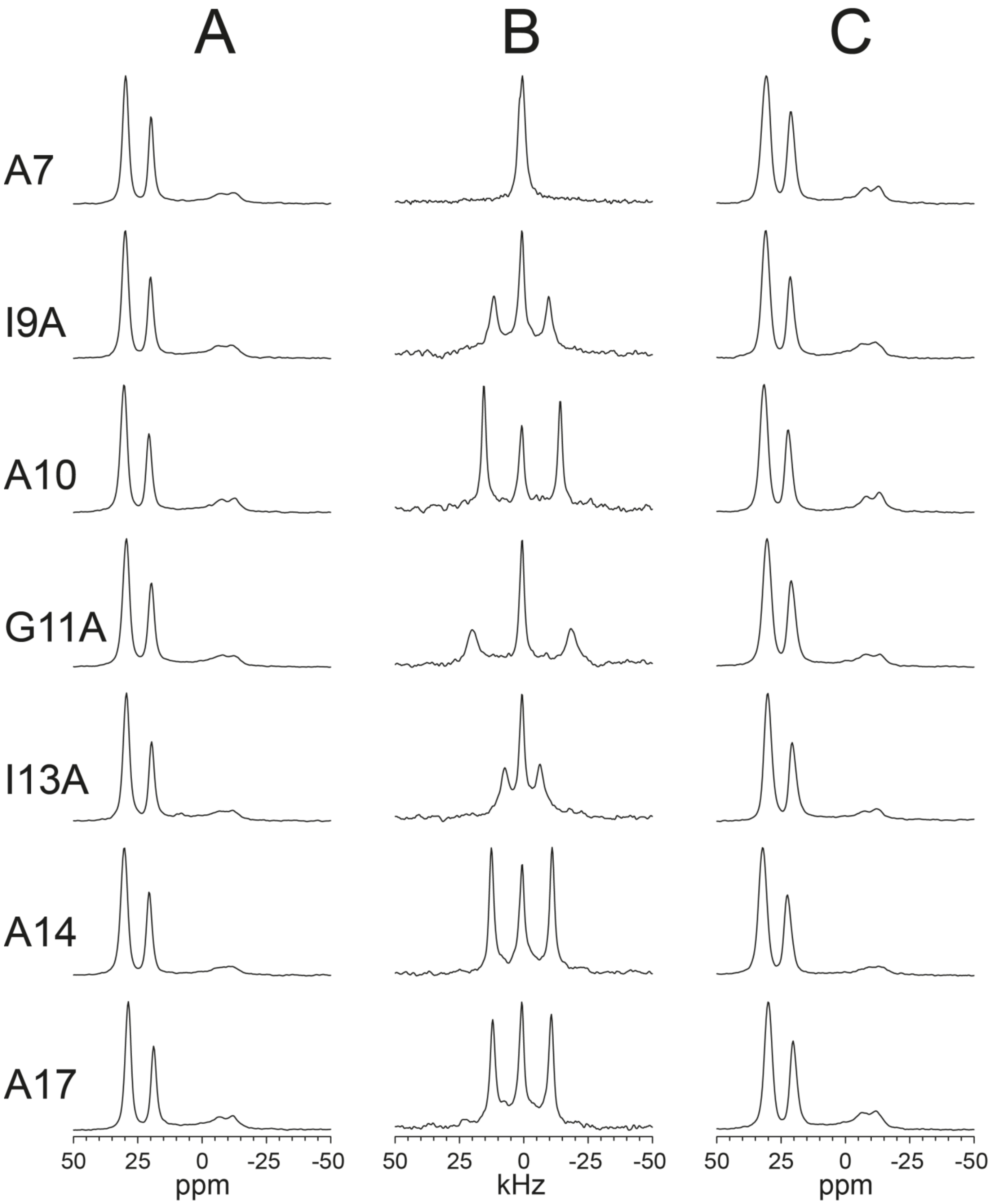

Figure S6. ^{31}P- and ^{2}H-NMR spectra of Ala-d_3 labeled MSI-103 in DLPC/lyso-LPC (2/1). (A) ^{31}P-NMR spectra before ^{2}H-NMR experiment. (B) ^{2}H-NMR spectra. (C) ^{31}P-NMR spectra after ^{2}H-NMR experiment.

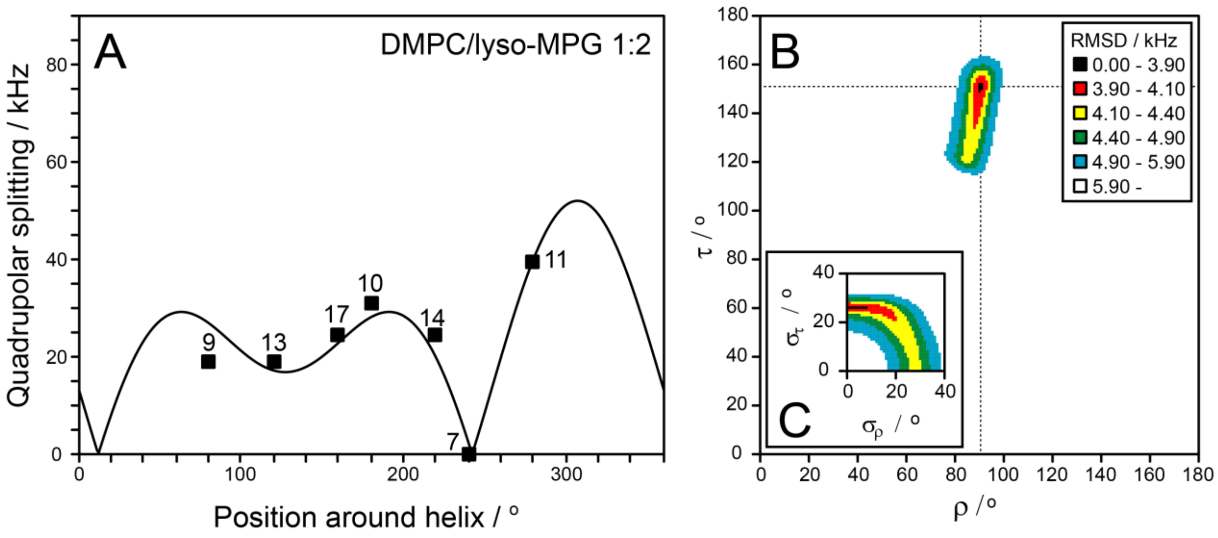

Figure S7. ^{2}H-NMR data analysis to determine the orientation of MSI-103 in DMPC/lyso-MPC (2/1) lipid bilayers at P/L=1/50. (A) The experimental data points fitted to helical curves with all data points projected to one turn around the helix. Numbers indicate the labeled position corresponding to each data point. (B) The RMSD for the best fit is a function of τ and ρ angles and is color-coded at each point. The best fit values are indicated by the intersection of the dotted lines. (C) The RMSD for the best fit as a function of σ_τ and σ_ρ. The color coding is the same in (B) and (C).

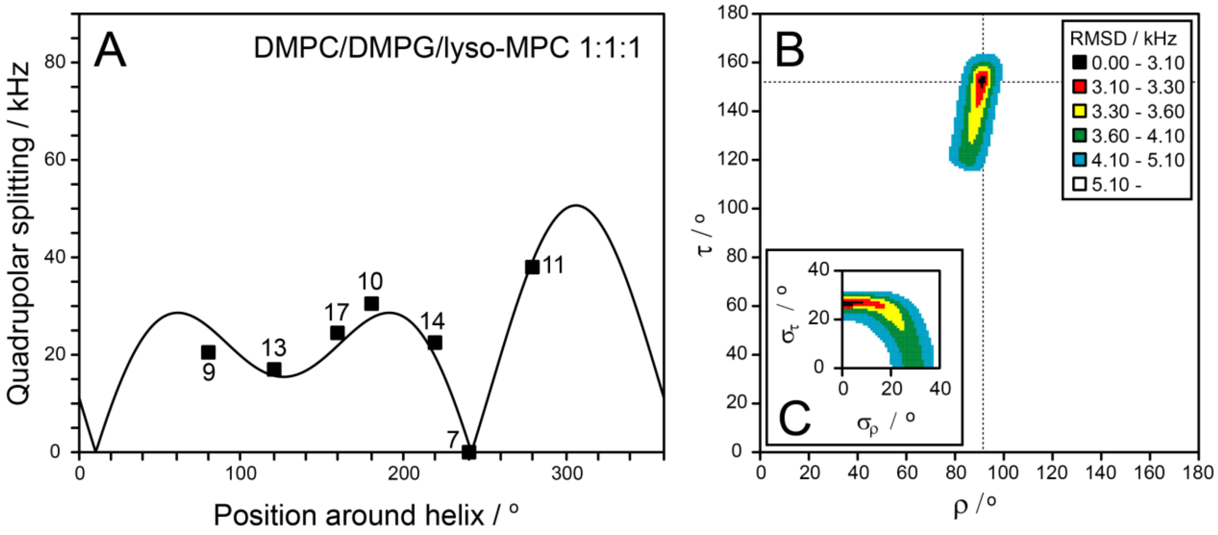

Figure S8. ^{2}H-NMR data analysis to determine the orientation of MSI-103 in DMPC/DMPG/lyso-MPC (1/1/1) lipid bilayers at P/L=1/50. (A) The experimental data points fitted to helical curves with all data points projected to one turn around the helix. Numbers indicate the labeled position corresponding to each data point. (B) The RMSD for the best fit is a function of τ and ρ angles and is color-coded at each point. The best fit values are indicated by the intersection of the dotted lines. (C) The RMSD for the best fit as a function of σ_τ and σ_ρ. The color coding is the same in (B) and (C).

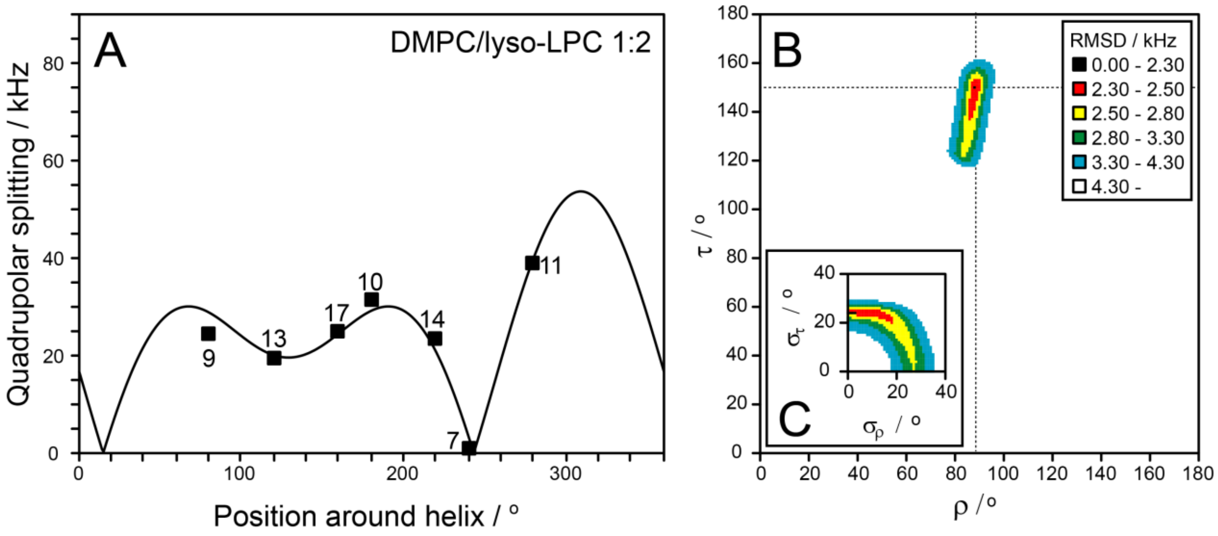

Figure S9. ^{2}H-NMR data analysis to determine the orientation of MSI-103 in DMPC/lyso-LPC (2/1) lipid bilayers at P/L=1/50. (A) The experimental data points fitted to helical curves with all data points projected to one turn around the helix. Numbers indicate the labeled position corresponding to each data point. (B) The RMSD for the best fit is a function of τ and ρ angles and is color-coded at each point. The best fit values are indicated by the intersection of the dotted lines. (C) The RMSD for the best fit as a function of σ_τ and σ_ρ. The color coding is the same in (B) and (C).

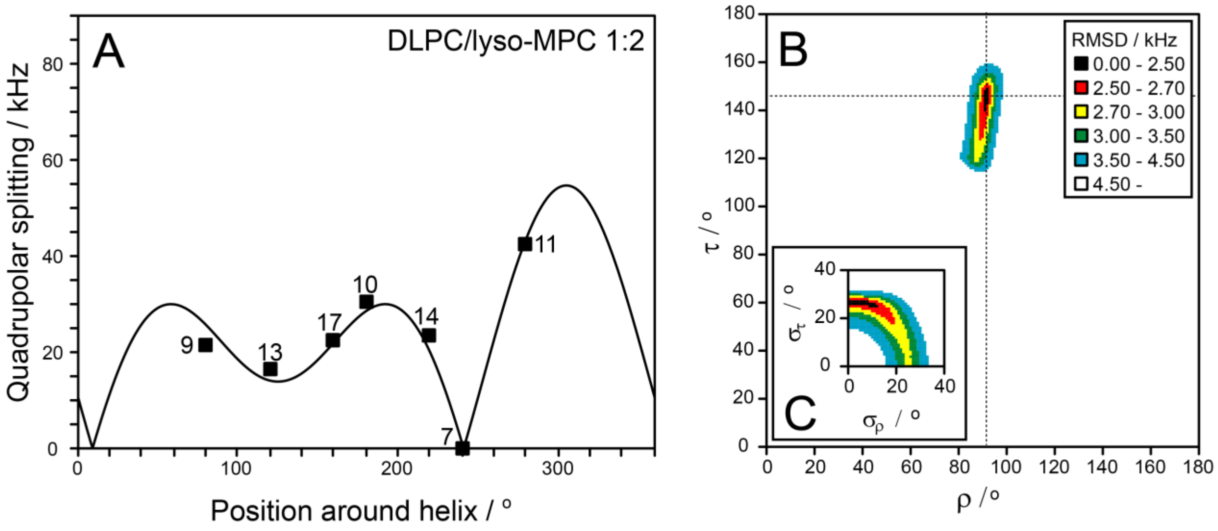

Figure S10. ^{2}H-NMR data analysis to determine the orientation of MSI-103 in DLPC/lyso-MPC (2/1) lipid bilayers at P/L=1/50. (A) The experimental data points fitted to helical curves with all data points projected to one turn around the helix. Numbers indicate the labeled position corresponding to each data point. (B) The RMSD for the best fit is a function of τ and ρ angles and is color-coded at each point. The best fit values are indicated by the intersection of the dotted lines. (C) The RMSD for the best fit as a function of σ_τ and σ_ρ. The color coding is the same in (B) and (C).

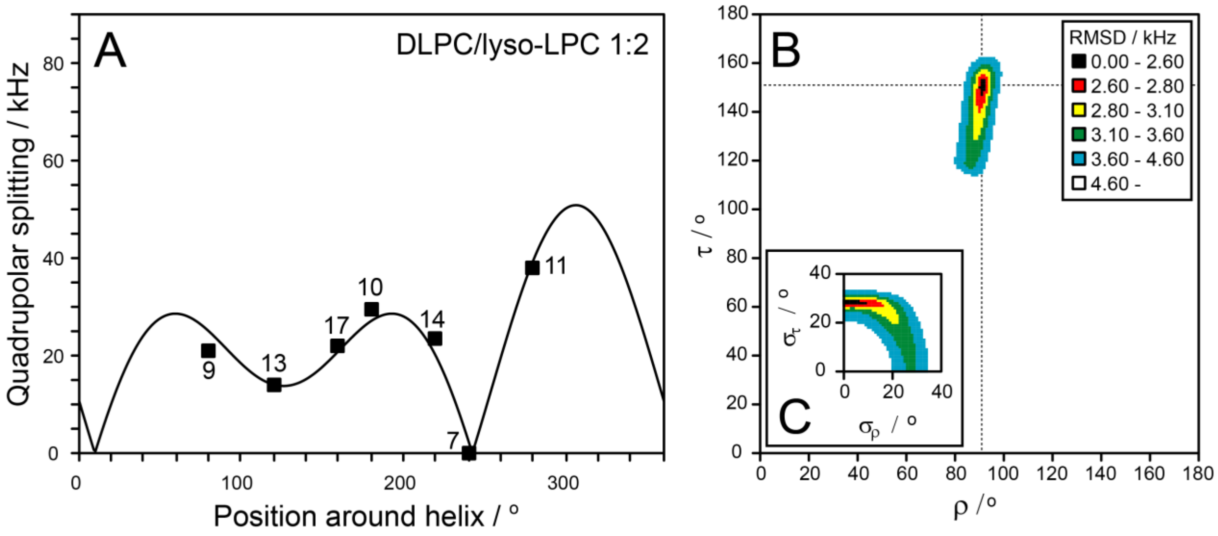

Figure S11. ^{2}H-NMR data analysis to determine the orientation of MSI-103 in DLPC/lyso-LPC (2/1) lipid bilayers at P/L=1/50. (A) The experimental data points fitted to helical curves with all data points projected to one turn around the helix. Numbers indicate the labeled position corresponding to each data point. (B) The RMSD for the best fit is a function of τ and ρ angles and is color-coded at each point. The best fit values are indicated by the intersection of the dotted lines. (C) The RMSD for the best fit as a function of σ_τ and σ_ρ. The color coding is the same in (B) and (C).
